# Imputation strategies for genomic prediction using nanopore sequencing

**DOI:** 10.1186/s12915-023-01782-0

**Published:** 2023-12-08

**Authors:** H. J. Lamb, L. T. Nguyen, J. P. Copley, B. N. Engle, B. J. Hayes, E. M. Ross

**Affiliations:** 1 Centre for Animal Science, Queensland Alliance for Agriculture and Food Innovation, The University of Queensland, St. Lucia, QLD 4067 Australia; 2 USDA, ARS, U.S. Meat Animal Research Centre, Clay Centre, NE 68933 USA

**Keywords:** Genotyping-by-sequencing, Skim-whole genome sequencing, Genotype imputation, Genomic prediction, Oxford Nanopore Technologies sequencing

## Abstract

**Background:**

Genomic prediction describes the use of SNP genotypes to predict complex traits and has been widely applied in humans and agricultural species. Genotyping-by-sequencing, a method which uses low-coverage sequence data paired with genotype imputation, is becoming an increasingly popular SNP genotyping method for genomic prediction. The development of Oxford Nanopore Technologies’ (ONT) MinION sequencer has now made genotyping-by-sequencing portable and rapid. Here we evaluate the speed and accuracy of genomic predictions using low-coverage ONT sequence data in a population of cattle using four imputation approaches. We also investigate the effect of SNP reference panel size on imputation performance.

**Results:**

SNP array genotypes and ONT sequence data for 62 beef heifers were used to calculate genomic estimated breeding values (GEBVs) from 641 k SNP for four traits. GEBV accuracy was much higher when genome-wide flanking SNP from sequence data were used to help impute the 641 k panel used for genomic predictions. Using the imputation package QUILT, correlations between ONT and low-density SNP array genomic breeding values were greater than 0.91 and up to 0.97 for sequencing coverages as low as 0.1 × using a reference panel of 48 million SNP. Imputation time was significantly reduced by decreasing the number of flanking sequence SNP used in imputation for all methods. When compared to high-density SNP arrays, genotyping accuracy and genomic breeding value correlations at 0.5 × coverage were also found to be higher than those imputed from low-density arrays.

**Conclusions:**

Here we demonstrated accurate genomic prediction is possible with ONT sequence data from sequencing coverages as low as 0.1 × , and imputation time can be as short as 10 min per sample. We also demonstrate that in this population, genotyping-by-sequencing at 0.1 × coverage can be more accurate than imputation from low-density SNP arrays.

**Supplementary Information:**

The online version contains supplementary material available at 10.1186/s12915-023-01782-0.

## Background

Genomic prediction is a method of using genome-wide DNA markers to assess the genetic merit of an individual and relies on the accurate genotyping of these DNA markers. As a result of their low cost [1] and impressive accuracy [2], SNP microarrays are currently the favoured method for genotyping in many species [3–5]. However, advances in next-generation sequencing technology have driven the cost of sequencing down significantly, making SNP genotyping from low-coverage sequence data, otherwise known as genotyping-by-sequencing (GBS), a potentially cost-competitive alternative. As the cost of GBS continues to decrease, the additional benefits of GBS, such as de novo variant discovery and structural variant genotyping, will likely cause a shift in genotyping preference. Two main GBS methods are currently used: reduced representation sequencing (RR-GBS) [6] and skim-GBS [7].

In RR-GBS, restriction enzymes are used to reduce genome complexity [6]. This results in higher sequencing coverages at certain loci for genotyping. However, RR-GBS increases the complexity of the lab protocols and has the potential to introduce sequencing bias [8]. At least 13 different protocols for RR-GBS have been developed, each of which differs slightly in the number and type of enzymes used [9]. The ability to decrease genome complexity means RR-GBS has been favoured for crop species with highly complex genomes, such as wheat [9, 10]. Furthermore, by increasing sequence coverage at regions for SNP genotyping, more accurate genotypes can be called without the need for genotype imputation. This means RR-GBS is also favoured for non-model species with limited prior genomic information [9]. Poland et al. [11] genotyped wheat varieties for less than USD $20 per sample using RR-GBS and predicted the cost of GBS will drop below USD $10 per sample.

Skim-GBS refers to calling genotypes from low coverage (< 1 × coverage) whole genome sequence data. Genotype imputation is often incorporated into skim-GBS pipelines to account for missing genotypes. Skim-GBS requires no additional laboratory steps, however, adds more computational complexity to account for the reduced likelihood of sampling all alleles at heterozygous loci in non-inbred species. Skim-GBS does not suffer from the same biases as RR-GBS methods however, the sequencing depth, marker density and population structure can impact imputation error rates significantly [12]. Imputation accuracies (*r*^2^) from high-density (HD) SNP array genotypes to whole genome SNP are regularly greater than 0.9 in humans [13] and livestock [14]. Similar imputation accuracies have been reported for GBS genotypes where sequencing coverage is greater than 2 × [15].

A number of genotype imputation packages have been developed and refined for accuracy and decreasing computational complexity. Popular genotyping packages include IMPUTE2 [16], Beagle4.1 [17], Beagle5.2 [18], Minimac3 [19] and Minimac4 [20]. Of these imputation packages, there is little difference in imputation accuracy, however, Browning et al. [18] demonstrated that Beagle5.0 was significantly faster than the other packages for large SNP panels.

Recently, a number of imputation packages targeted specifically at GBS data have been developed. These second-generation imputation packages have been developed to handle larger SNP panels and use genotype likelihoods or physical linkage information from the sequence reads themselves, in order to maximise the use of available information for imputation. Examples of these packages include QUILT [21] and Genotype Likelihoods IMputation and PhaSing Method (GLIMPSE) [22]. QUILT is a rapid genotype imputation and phasing package developed specifically for GBS and uses sequence alignment files as input. QUILT uses a two-step Gibbs sampling method to ensure linear increases in computational complexity with increasing reference size and SNP density. Imputation accuracies greater than 0.9 have been achieved using QUILT with Oxford Nanopore Technologies (ONT) data at 0.5 × sequencing coverage while comparatively, GLIMPSE achieved an imputation accuracy of 0.68 using the same data [21]. GLIMPSE uses genotype likelihoods calculated during the prior genotyping step [22]. Unlike other earlier imputation methods which also use genotype likelihoods (e.g., Beagle 4.1), GLIMPSE is able to handle much larger SNP panels consisting of thousands of haplotypes [22].

To date, short-read sequencing methods, such as Illumina, have been favoured for GBS due to their well-characterised error profile, high throughput and competitive cost. These platforms produce highly accurate short (200–400 bp) DNA reads [23]. Long-read sequencing platforms such as Pacific Biosciences and ONT are comparatively new technologies and could provide an alternative platform for GBS. These platforms produce long DNA reads (2 kbp–2 Mbp) that can map more accurately to reference genomes; however, they have historically had higher individual nucleotide error rates. In the case of ONT, the error rate is reportedly between 2.7 and 7% [24, 25], although this is improving rapidly due to advances in base calling algorithms. ONT sequencing is, however, the only sequencing platform to currently offer portable sequencing through their MinION and PromethION P2 sequencers. This feature may be a great advantage in the extensive livestock industries [26].

Given the current price of ONT sequencing, to compete with SNP array technology, sequencing coverages less than 0.5 × for a human-sized genome will be necessary to allow adequate multiplexing. This raises the question; can we accurately predict genetic merit from less than 0.5 × ONT sequencing coverage? To add additional complexity, turnaround time must also be considered when evaluating pipelines, as lengthy posterior bioinformatics would undermine the advantage of in situ genotyping.

Our objective was to investigate the optimum imputation strategy for skim-GBS from ONT sequence data for the purpose of in situ genomic prediction. We evaluated the performance of Beagle5.2, QUILT and GLIMPSE using five different SNP reference panels and five different sequencing coverages. We hypothesised that genomic prediction accuracy would be improved if all available SNP in sequence sets were used for imputation, not only the subset used in the genomic prediction. That is, multiple SNP flanking the target SNP should give additional information for imputation from sequence data.

## Results

### Genotyping and imputation

ONT sequence data from 62 samples sequenced in Hayes et al. [27] on a MinION was subsampled down to 2 × , 1 × , 0.5 × , 0.1 × and 0.05 × sequencing coverage (Additional File 1: Table 1). Samples were from 48 unique animals (14 of which were sampled twice at two separate time points) from two predominantly *Bos indicus* breeds (Brahman and Droughtmaster). The reads were aligned to the *Bos taurus* reference genome ARS-UCDv1.2 [28] using Minimap2 [29].

**Table 1:** Details of the SNP panels using the different minor allele frequency (MAF) filters

SNP panel name	MAF cut-off	Number of animals	Number of SNP	Mean distance between markers (bp)
Bovine HD SNP	NA	1208	641,163	4212
No MAF filter	0	1208	48,203,338	56
MAF > 0.1	0.1	1208	15,000,408	180
MAF > 0.2	0.2	1208	9,544,194	283
MAF > 0.3	0.3	1208	4,575,736	590

*SNP* single nucleotide polymorphisms, *MAF* minor allele frequency

Two different genotyping methods were used to genotype the samples at 48,203,338 loci across the genome. These SNP were derived from the 1000 Bull Genomes project [30], with the SNP from this project filtered to those segregating in Brahman, Droughtmaster, Santa Gertrudis and crossbred animals (1208 animals from 1000 Bull genomes run 8), which are the most relevant animals to Australia’s northern beef industry. The first genotyping method used a minimum allele count (MAC) based on sequencing depth as described in Lamb et al. [31]. The second method, hereafter referred to as the Q-score method, used base qualities to assign genotype likelihoods using the original GATK method [32] and also incorporated a methylation masking approach to avoid calling genotypes at potential methylation sites. This worked by identifying the potential methylation positions in the SNP loci and only calling SNP on the non-methylated strand.

**Fig. 1:**
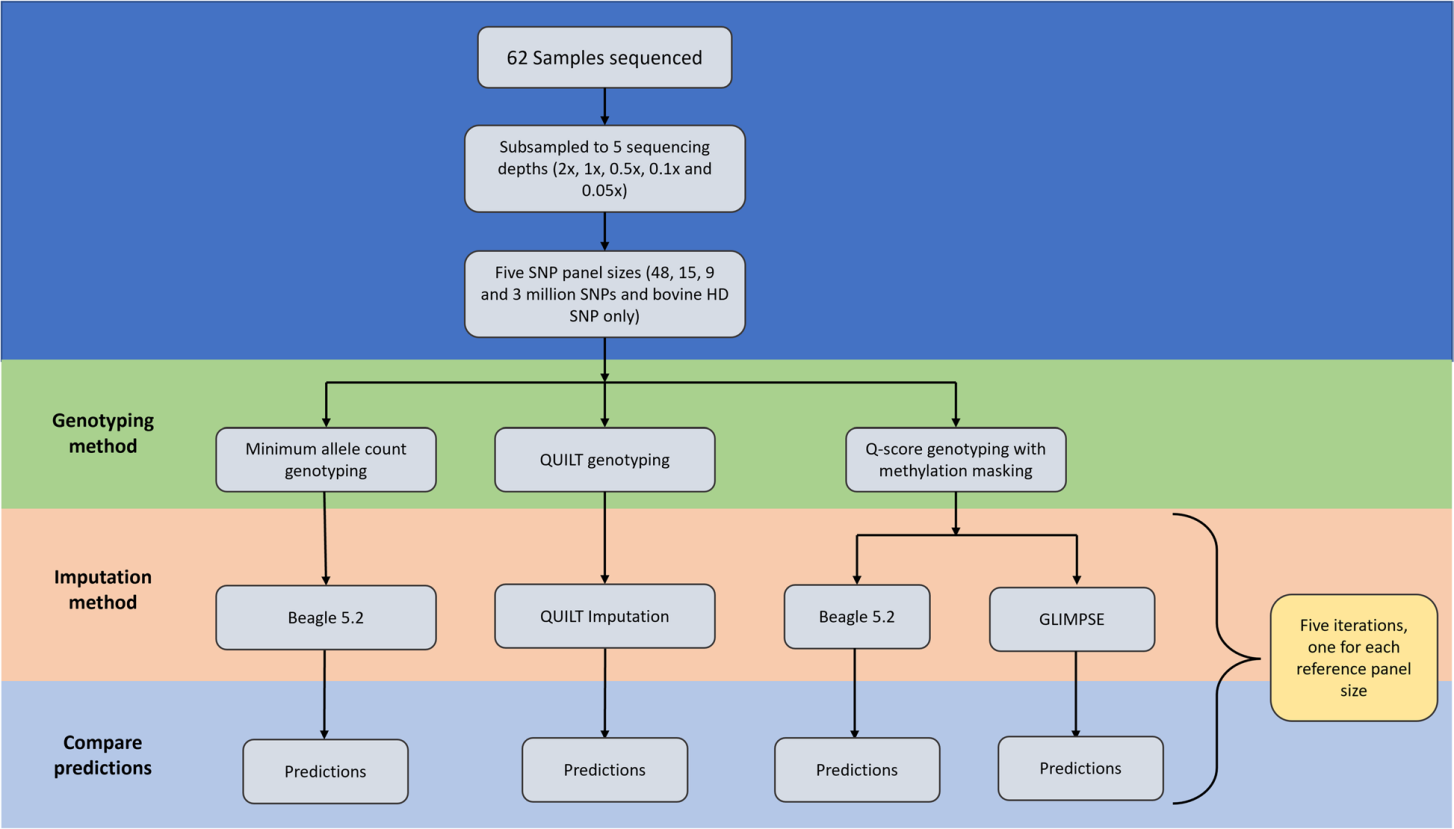
Flowchart of the genotyping and imputation methods used to generate genomic estimated breeding values from the Oxford Nanopore Technologies sequence data

Three methods were used to impute missing SNP from the genotype calls (Fig. 1). Genotype likelihoods from the Q-score method were used to impute loci using GLIMPSE, while the genotype calls from the MAC and Q-score methods were used to impute loci using Beagle5.2. The final method used QUILT to genotype and impute the loci in one step from the alignment files.

Five different SNP reference panels were used for imputation (Table 1). The animals in these reference panels were the 1208 animals described above (Additional file 1: Table S2 and Fig. S1). The first SNP panel consisted of only the 641,163 SNP that overlapped with the bovine HD SNP array (Illumina, Inc., San Diego USA). The second SNP panel contained the entire 48,203,338 SNP from the 1000 Bull Genomes project that were segregating in northern Australian cattle breeds. The three remaining SNP panels were a subset of this larger panel and were created using minor allele frequency (MAF) filters of 0.1, 0.2 and 0.3. Minor allele frequency filters were used to reduce the size of the SNP panels to increase the speed of imputation. All 641,163 SNP from the bovine HD SNP array used for calculating genomic breeding values (described below) were also retained in the reference SNP panels.

### Correlations of genomic predictions

The imputed genotypes at the 641,163 SNP loci that corresponded to the bovine HD array were used to calculate genomic estimated breeding values (GEBVs) for four traits, body weight (BW), hip height (HH), *corpus luteum* score (CL score) and body condition score (BCS). The SNP effects used to calculate the GEBVs were derived from 28,000 animals with genotypes and phenotypes as described in Hayes et al. [33] and Lamb et al. [31]*.* Correlations between the ONT GEBVs and GEBVs derived from the 35 K GGP TropBeef SNP array (Neogen, Lansing, MI) genotypes imputed to the bovine HD array using Fimpute [34] and estimated using the same SNP effects [33] were calculated.

When genotypes for each animal were imputed using only SNP from the bovine HD array in the reference SNP panel (641,163 SNP), correlations between the ONT GEBVs and GEBVs from the 35 k array were between 0.74 and 0.92 for sequencing coverages less than 1 × , using MAC-Beagle5.2 and Q-score-Beagle5.2. However, GEBV correlations using QUILT and GLIMPSE with the HD size reference panel were greater than 0.9 for all four traits at 0.5 × sequencing coverage.

**Fig. 2:**
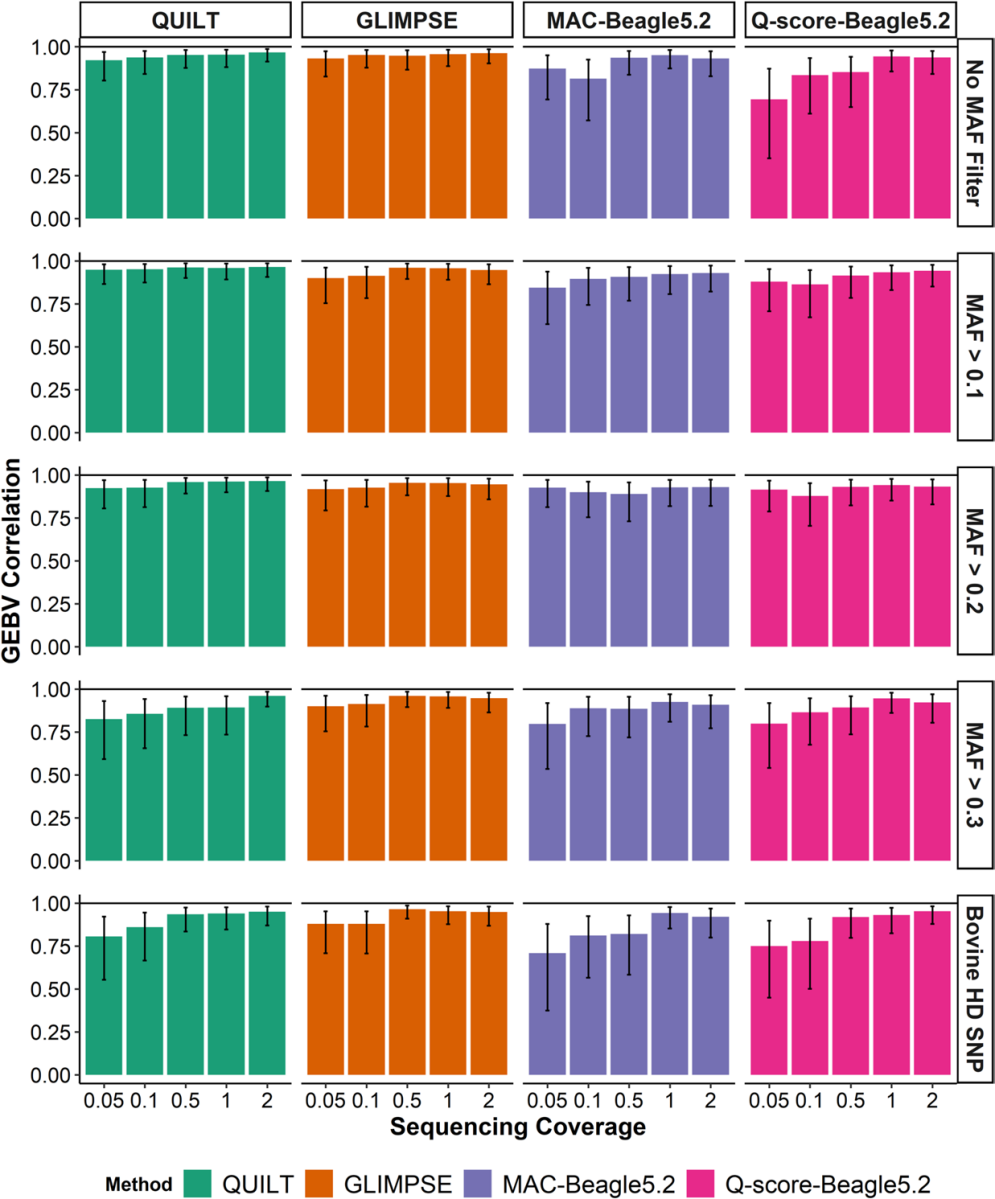
Correlations between body weight (BW) genomic estimated breeding values (GEBV) derived from 35 k SNP array genotypes and BW GEBVs derived from Oxford Nanopore Technologies (ONT) data. ONT GEBVs were imputed using four different imputation strategies and across five sequencing coverages. Labels at the top of the figure indicate the imputation method used starting from left to right with GLIMPSE [22], minor allele count (MAC) genotyping with Beagle5.2 [18], quality score (Q-score) genotyping with Beagle5.2 and QUILT [21]. SNP reference panel size is indicated by the minor allele frequency (MAF) filter on the right-hand side in descending order of size from top to bottom. The largest panel had 48,203,338 SNP and was referred to as the No MAF filter panel, while the smallest panel was referred to as the bovine high density (HD) SNP which had only the 641 k SNP used to calculate the GEBVs. Error bars indicate 95% confidence of the Pearson correlation

When the 48 million flanking SNPs were used to help impute the 641 k SNP genotypes from the sequence data, correlations using QUILT and GLIMPSE were much higher (greater than 0.82 for 0.05 × and greater than 0.93 for 2 × sequence coverage), and higher than the other two imputation methods, particularly at low coverages (Fig. 2 and Additional file 1: Fig. S2–S4). The correlations remained similar using the next two largest SNP reference panels (MAF > 0.1 and MAF > 0.2), however, decreased using the smallest SNP panel to between 0.77 and 0.88.

Although all imputation methods performed well at sequencing coverages greater than 0.5 × , only QUILT and GLIMPSE maintained high GEBV correlations at sequencing coverages below 0.5 × . In particular, QUILT maintained very high GEBV correlations for all sequencing coverages when using the second largest imputation reference panel (MAF > 0.1).

No difference between GEBV correlations for the two Beagle5.2 imputation methods was observed. Both methods demonstrated a similar decrease in correlations for sequencing coverages below 0.5 × . Even using the largest SNP panel for imputation, the correlations between the ONT BW GEBVs and 35 k SNP array BW GEBVs at 0.1 × sequencing coverage were 0.81 and 0.83 for the two Beagle5.2 methods, compared to 0.94 and 0.95 using QUILT and GLIMPSE, respectively.

### Genomic prediction bias

**Fig. 3:**
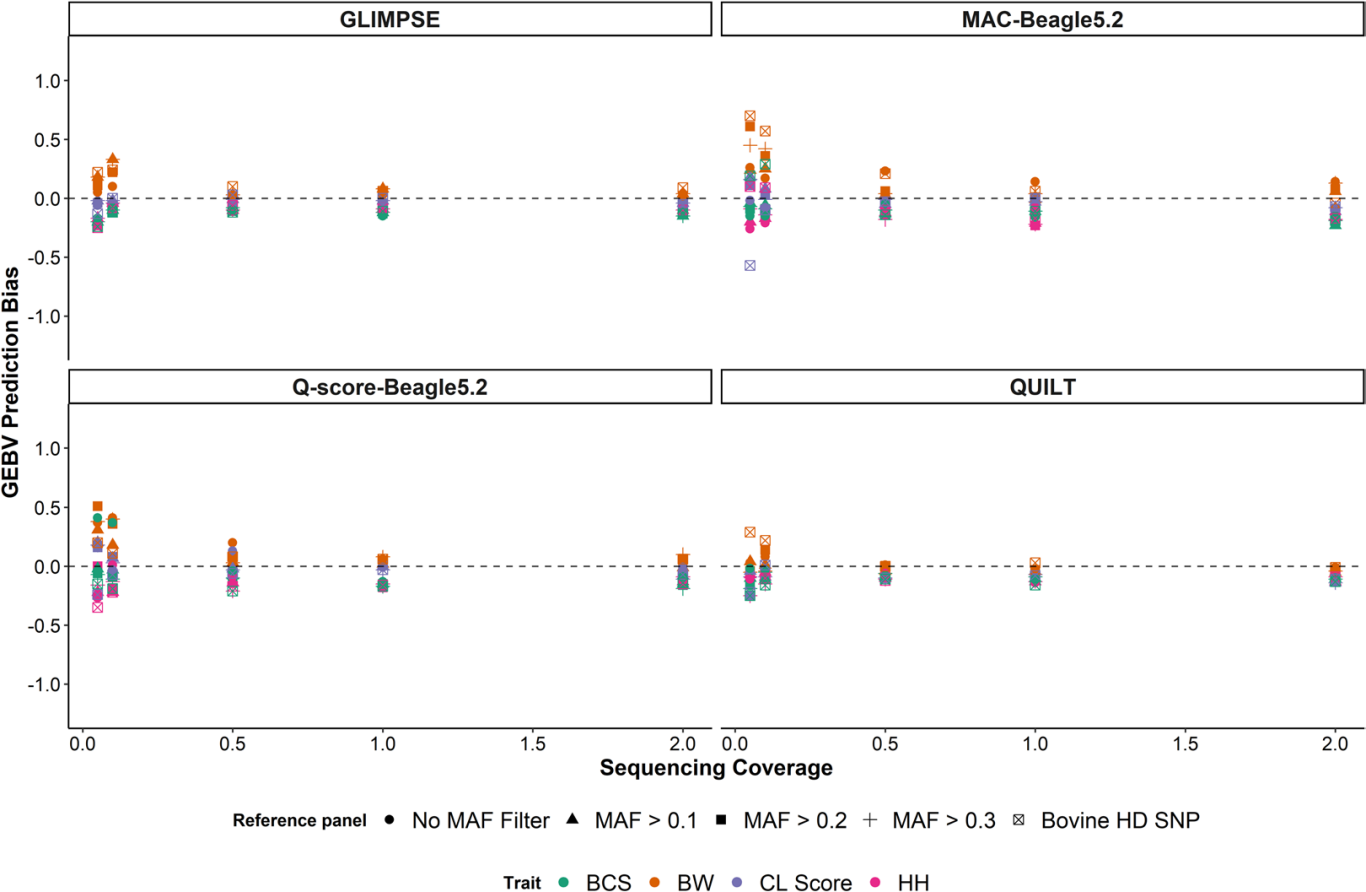
Genomic prediction bias, defined as ${\beta }_{1}-1$, where ${\beta }_{1}$ is the regression coefficient of the 35 k SNP array genomic estimated breeding values ~ Oxford Nanopore Technologies derived genomic estimated breeding values, for the four different imputation approaches across the sequencing coverages for four traits: body weight (BW), body condition score (BCS), *corpus luteum* score (CL score) and hip height (HH). Labels at the top of each figure indicate the imputation method used starting with GLIMPSE [22], minor allele count (MAC) genotyping with Beagle5.2 [18], quality score (Q-score) genotyping with Beagle5.2 and QUILT [21]. Prediction bias was also calculated across five different SNP reference panel sizes which were created using minor allele frequency (MAF) filters from whole genome sequence SNP. The smallest SNP reference panel, the bovine high definition (HD) SNP, had only the 641 k SNP used to calculate the GEBVs

The regression coefficient of the 35 k SNP array GEBV on ONT GEBV was used to measure prediction bias for each imputation strategy. Predictions using QUILT and GLIMPSE had much less bias than predictions derived from the other two methods (Fig. 3). For sequencing coverages greater than 0.5 × , the bias using QUILT and GLIMPSE was low across all traits and SNP reference panels. Below 0.5 × prediction bias was more variable across traits and SNP panel sizes.

For both of the Beagle imputation methods, significantly more variation in prediction bias was observed across traits and SNP panel size, with both SNP reference panel size and sequencing coverage having a negative directional effect on prediction bias (*P* < 0.05).

Across the four traits, no difference in prediction bias was observed except for in BW (*P* < 0.05). Using all four methods BW prediction bias was consistently higher than the other three traits. This was particularly evident in the MAC-Beagle5.2, Q-score-Beagle5.2 and GLIMPSE predictions, as well as at low sequencing coverages.

### Imputation compute time

**Fig. 4:**
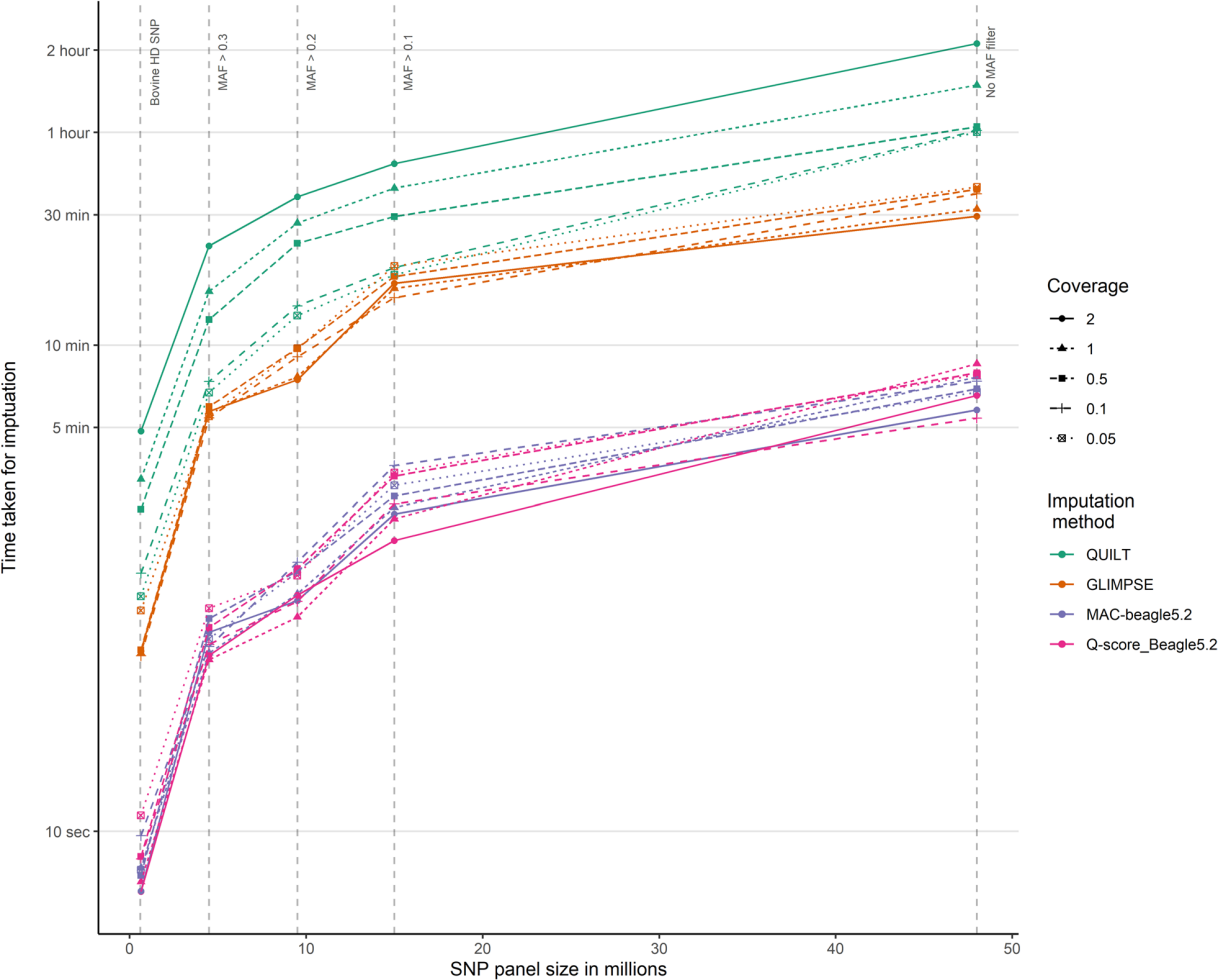
Mean time taken to impute the genotypes of each animal from Oxford Nanopore Technologies sequence data using four different imputation methods. Genotypes were imputed using five SNP reference panels created using minor allele frequency (MAF) filters

The time taken to impute genotypes for each animal, using 8 threads with 400 Gb of memory on the University of Queensland high-performance compute cluster, was recorded in order to compare the speed of each method (Fig. 4). Both methods using Beagle5.2 were faster than QUILT and GLIMPSE. Imputation time decreased linearly with the size of the reference SNP panel. At coverages greater than 0.1 × , QUILT was the slowest imputation method across all SNP panel sizes by a large margin. While at 0.1 × and 0.05 × sequencing coverage the imputation times for QUILT and GLIMPSE were similar, except when using the largest SNP reference panel.

For GLIMPSE and both Beagle5.2 methods, sequencing coverage had a relatively small effect on the imputation time compared to reference panel size. While for QUILT both coverage and SNP panel size had a significant effect on imputation times (*P* < 0.05).

**Fig. 5:**
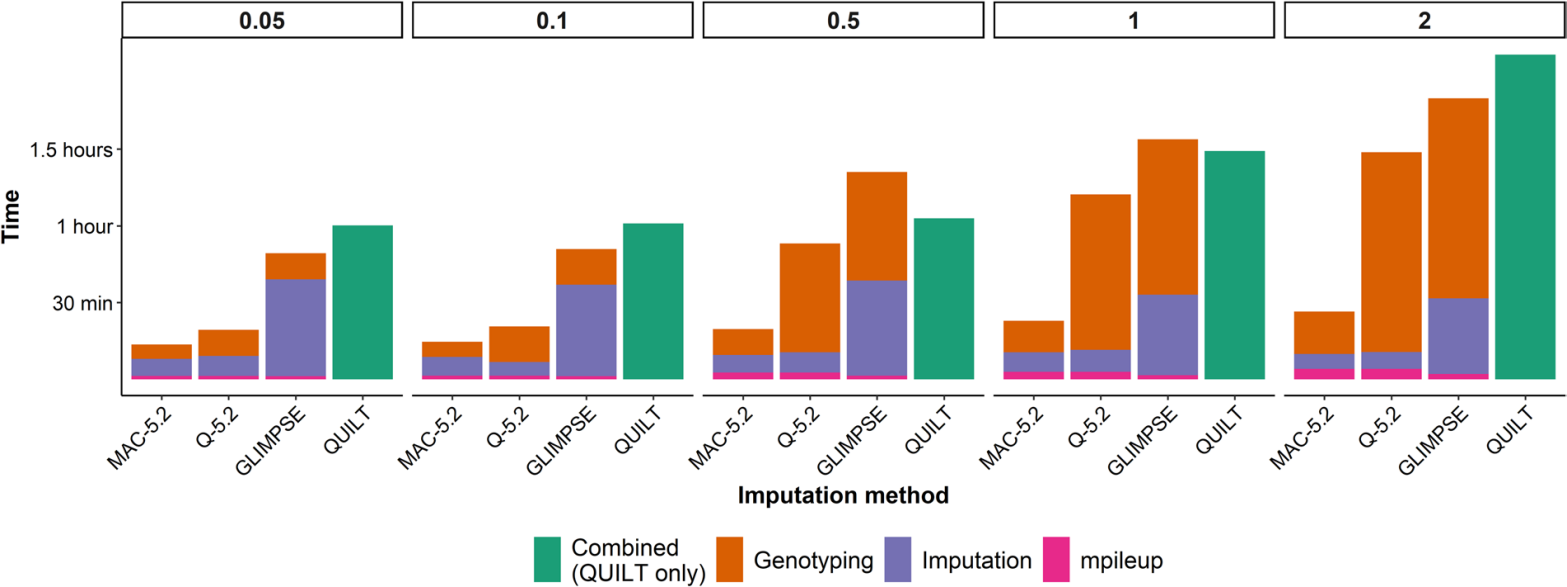
Average time taken for genotyping and imputation of all 48 million SNP in the unfiltered SNP panel from sequence alignment file using the four different methods and the five sequencing coverages. The imputation method QUILT genotyped and imputed SNP in one iteration while the other three methods used base pair position pileups to genotype and then impute missing SNP

The time taken to generate the sequence pileup and genotype loci using the MAC and Q-score methods was also recorded for the largest SNP panel. As QUILT effectively performs these tasks during imputation to compare the overall time of each method, these times were also included (Fig. 5). Taking into account these additional steps, the MAC-Beagle5.2 method was significantly faster than the alternative methods, particularly at higher sequencing coverages. At 2 × sequencing coverage, MAC-Beagle5.2 was overall 2.3 times faster than the next fastest method, which was Q-score-Beagle5.2. This decreased to 1.1 times faster at 0.05 × sequencing coverage; however, this was still 1.8 and 2.1 times faster than GLIMPSE and QUILT.

### Comparison to HD SNP array

**Fig. 6:**
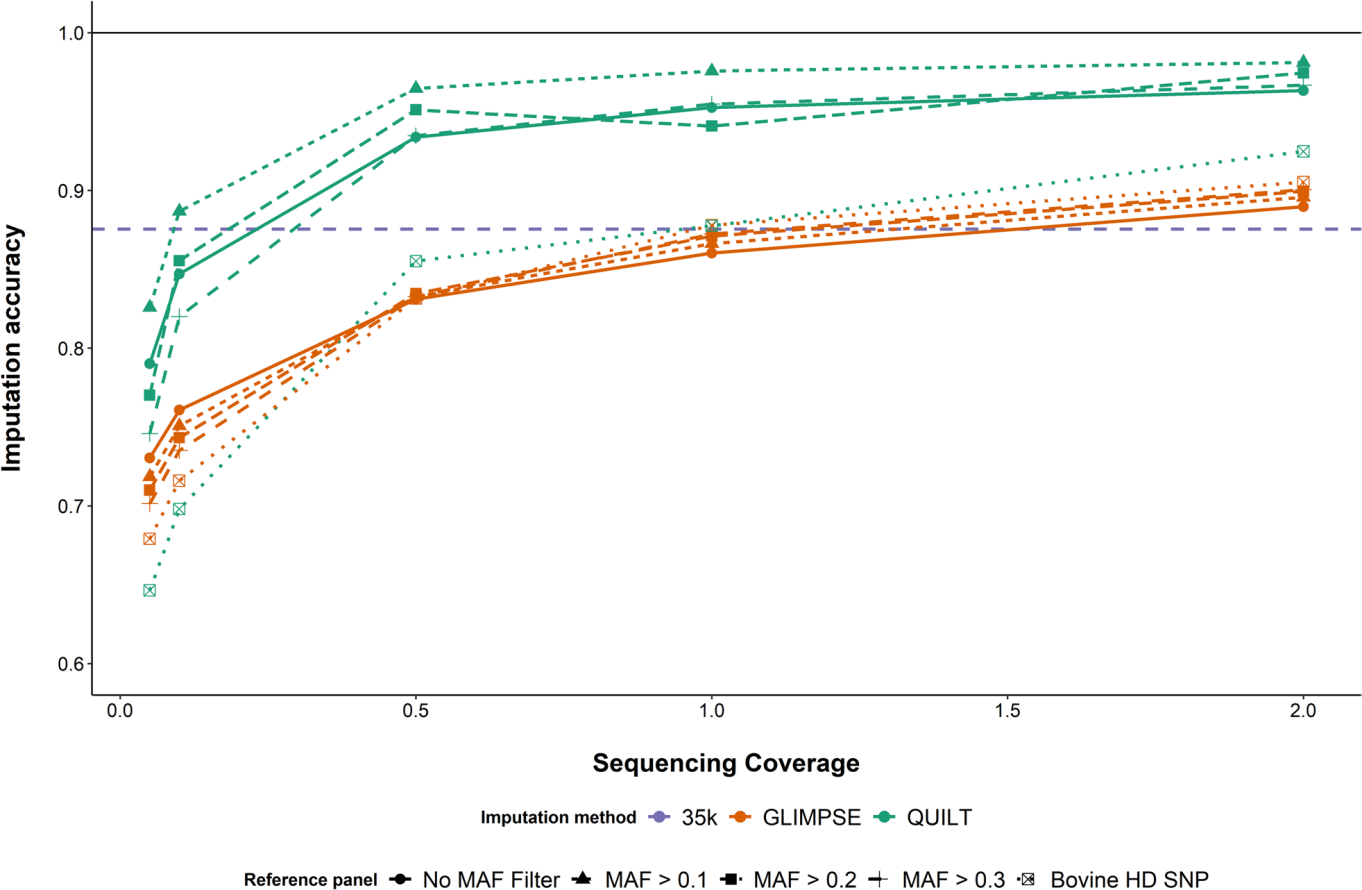
Imputation accuracy for genotypes derived from low-coverage Oxford Nanopore Technologies (ONT) sequence data imputed using QUILT and GLIMPSE and compared to bovine HD SNP array genotypes. ONT genotypes were imputed across five different sequencing coverages and using five different imputation reference panels. The imputation accuracy of genotypes imputed from the low-density SNP array to the HD array density is illustrated by the green dashed line

There is some imputation inaccuracy even when imputing from 35 k array genotypes up to the bovine HD array genotypes used in our prediction equations. So, an interesting question is, how do low-coverage ONT GEBVs compare to GEBVs from bovine HD array genotypes, and do they perform better than HD genotypes imputed from 35 k array genotypes? A subset of 19 animals were also genotyped on the Illumina bovine HD SNP array. These animals were used to investigate the accuracy of genotyping-by-sequencing with ONT data using our two best-found imputation methods (QUILT and GLIMPSE), as compared to genotyping and imputing from low-density SNP arrays. Genotyping accuracy was calculated and GEBVs for the four traits were also derived. The imputation accuracy was calculated for QUILT and GLIMPSE by comparing the imputed genotypes at the HD SNP array loci to the genotypes from the bovine HD SNP array. Genotypes imputed using QUILT had the highest accuracy across all sequencing coverage and imputation reference panels, except at 0.1 × and 0.05 × sequencing coverage with the smallest imputation reference panel (Fig. 6). The highest imputation accuracy achieved was 0.98 at 2 × sequencing coverage with the second largest imputation reference panel. At 0.5 × sequencing coverage, the highest accuracy using QUILT was 0.96, while using GLIMPSE, the highest accuracy was 0.8. In contrast, the imputation accuracy of the 35 k array compared to the HD SNP array was found to be 0.88. Imputation reference panel size was found to have less of an effect on imputation accuracy for GLIMPSE than QUILT. At 0.5 × sequencing coverage and greater, the imputation accuracy using QUILT was greater than the imputation accuracy from the low-density SNP array when imputed to the HD SNP array, except using the smallest imputation reference panel. For all sequencing coverages, QUILT with the second largest imputation reference panel (MAF > 0.1) had the highest imputation accuracy.

**Fig. 7:**
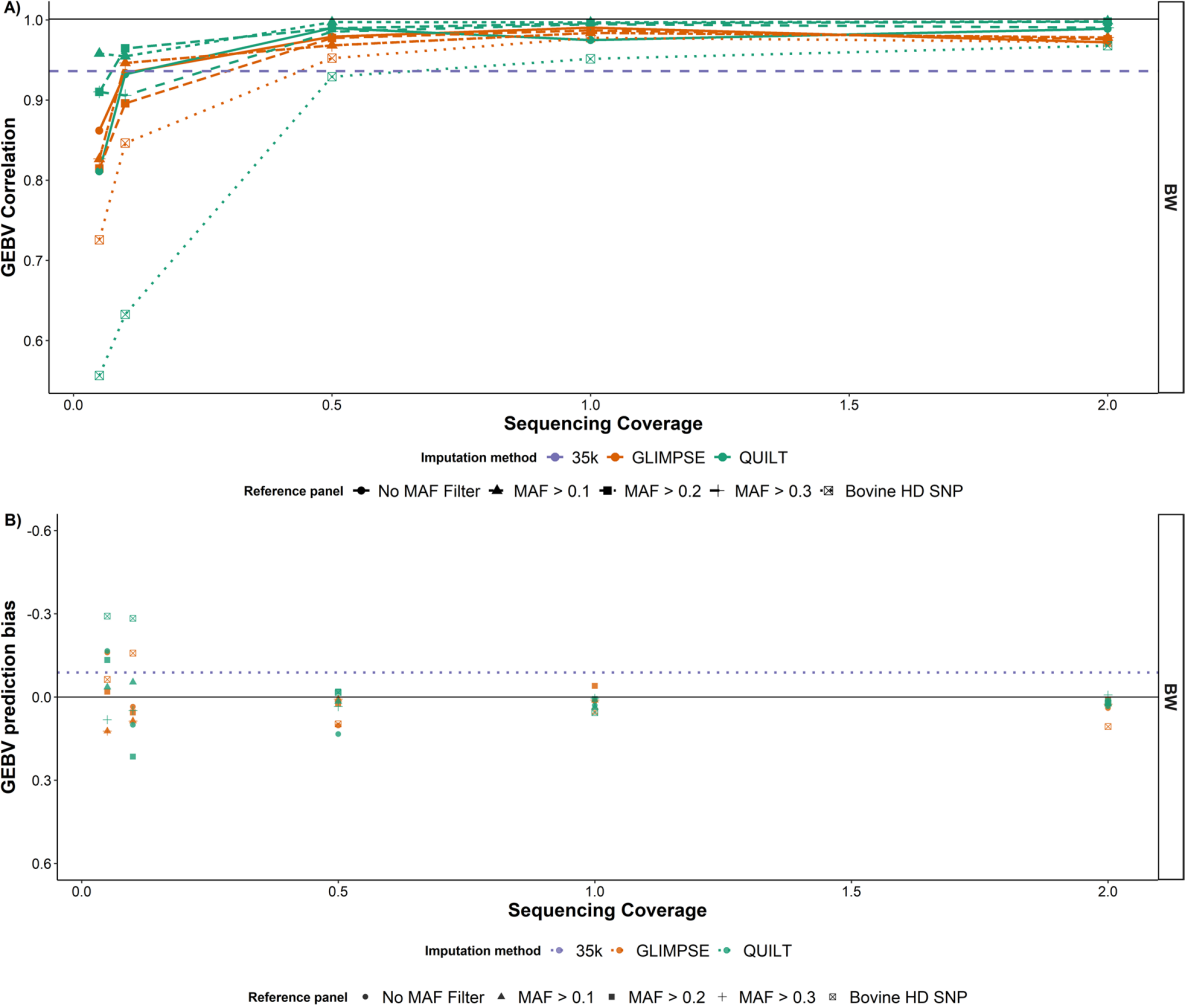
**A** Correlations between genomic estimated breeding values (GEBVs) derived from Oxford Nanopore Technologies (ONT) sequence data and GEBVs derived from bovine HD SNP array genotypes for body weight (BW). ONT-derived GEBVs were imputed using QUILT and GLIMPSE and calculated across five coverages and five SNP panels. The different SNP reference panels were created using minor allele frequency (MAF) filters to reduce the size of the panels down from whole genome sequence SNP. The largest panel had 48,203,338 SNP and was referred to as the No MAF filter panel, while the smallest panel was referred to as the bovine high definition (HD) SNP panel and featured only the 641 k SNP used to calculate the GEBVs. SNP array genotypes were from the Illumina bovine HD SNP array. The correlation for each trait between GEBVs calculated from the 35 k GGP SNP array imputed to 700 k and GEBVs calculated from the Illumina bovine HD SNP array are indicated by the dashed line. The colour of each bar indicates how well the ONT derived GEBV accuracies compare to the 35 K SNP array accuracies. Error bars indicate 95% confidence interval of the Pearson correlation. **B** Genomic prediction bias for body weight (BW), defined as ${\beta }_{2}-1$, where ${\beta }_{2}$ is the regression coefficient of the bovine HD SNP array genomic estimated breeding value (GEBV) ~ Oxford Nanopore Technologies GEBV derived using QUILT and GLIMPSE. The prediction bias of the HD SNP array GEBVs ~ 35 k SNP array GEBVs are displayed for each trait by the dotted lines, where the colour of the line corresponds to the colour of the trait in the figure legend

Next, correlations between ONT GEBVs (imputed using QUILT and GLIMPSE) and the HD SNP array GEBVs were calculated. For sequencing coverages as low as 0.5 × , the ONT GEBVs had higher correlations than the 35 k SNP array GEBVs (Fig. 7 and Additional file 1: Fig. S5–S7). For 0.1 × coverage, the QUILT GEBV correlations were not different to the 35 k SNP array GEBV correlations (*P* > 0.05). While below 0.1 × sequencing coverage the QUILT GEBV correlations were worse than the 35 k SNP array correlations for some, but not all of the traits and reference panel designs. Although QUILT performed better than GLIMPSE for the majority of sequencing coverages and larger imputation reference panel sizes, the GEBV correlations decreased significantly when using the smallest imputation reference panel size at low coverages. For example, the GEBV correlations using QUILT with the Bovine HD SNP reference panel at 0.1 × were between 0.63 and 0.69 for the four different traits.

**Fig. 8:**
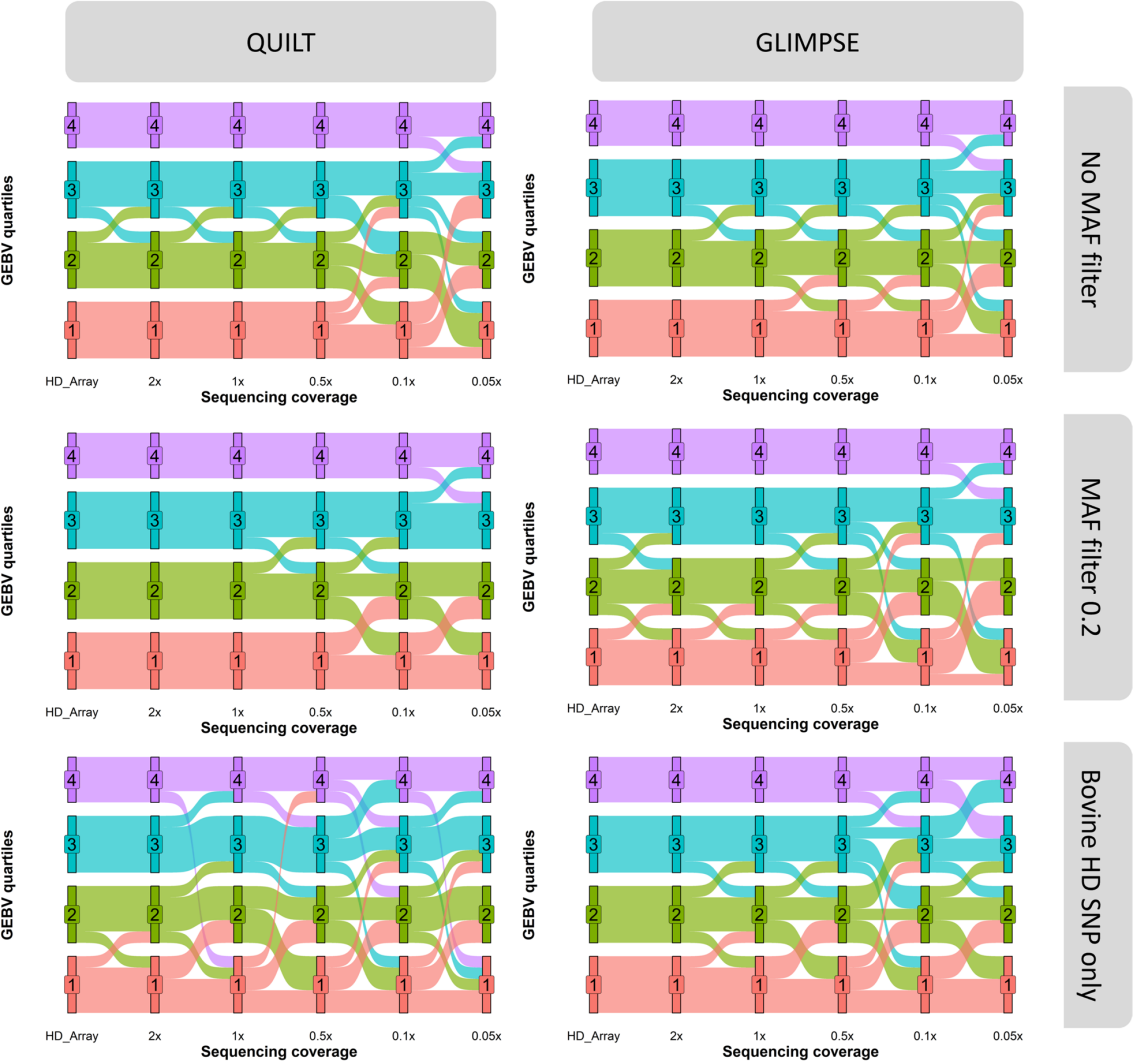
Change in body weight genomic estimated breeding values (GEBV) quartile rankings between the HD SNP array GEBVs and GEBVs derived from five different Oxford Nanopore Technologies (ONT) sequencing coverages. ONT GEBVs were derived using either GLIMPSE or QUILT for genotype imputation. Three different imputation reference panels were used: The first reference panel included all 48 million SNP; the second reference panel used a minor allele frequency (MAF) filter of > 0.2 and had 9.5 million SNP. The third reference panel included only the 700,000 SNP in the bovine HD SNP array

The regression coefficients of the bovine HD SNP array GEBVs ~ ONT GEBVs (imputed using QUILT and GLIMPSE) were randomly centred around 1, indicating no systematic prediction bias. Sequencing coverages greater than or equal to 0.5 × had little prediction bias (Fig. 8), while at sequencing coverages below 0.5 × prediction bias increased randomly in both directions, except when using QUILT with the second largest reference panel (MAF > 0.1). For all sequencing coverages, QUILT in combination with the second largest reference panel had less prediction bias than the 35 k SNP array predictions. At 0.5 × , the regression coefficient of the HD SNP array GEBVs ~ QUILT ONT GEBVs was comparable to the regression coefficient of HD SNP array GEBVS ~ 35 k SNP array GEBVs (BW = 0.91, HH = 0.88, CL score = 1, BCS = 0.95). Both SNP panel size and trait also had no correlation with prediction bias.

A major benefit of using GEBVs from a producer’s perspective is the ability to quantitatively rank animals for a particular trait based on their genetic merit. To investigate the ability for low-coverage ONT sequence data to accurately rank animals, we compared the rankings of animals based on ONT GEBVs across coverages to GEBVs calculated from the bovine HD array genotypes of 19 animals. Minimal re-ranking of animals was observed down to a coverage of 0.5 × . Below 0.5 × , ONT GEBVs regressed towards the mean, creating more re-ranking of animals and less apparent difference between the performance of animals (Fig. 8). However, animals were still clearly distinguishable into quartiles and the majority of re-ranking was between animals with extremely similar HD SNP array GEBVs. ONT GEBVs derived using QUILT had less re-ranking than ONT GEBVs derived from GLIMPSE for the larger imputation reference panels, while for the smallest imputation reference panel QUILT had significantly more re-ranking than GLIMPSE. Overall, the least re-ranking across sequencing coverages was observed when using the second largest imputation reference panel (MAF > 0.1) and QUILT for imputation.

### Genotype concordance

**Fig. 9:**
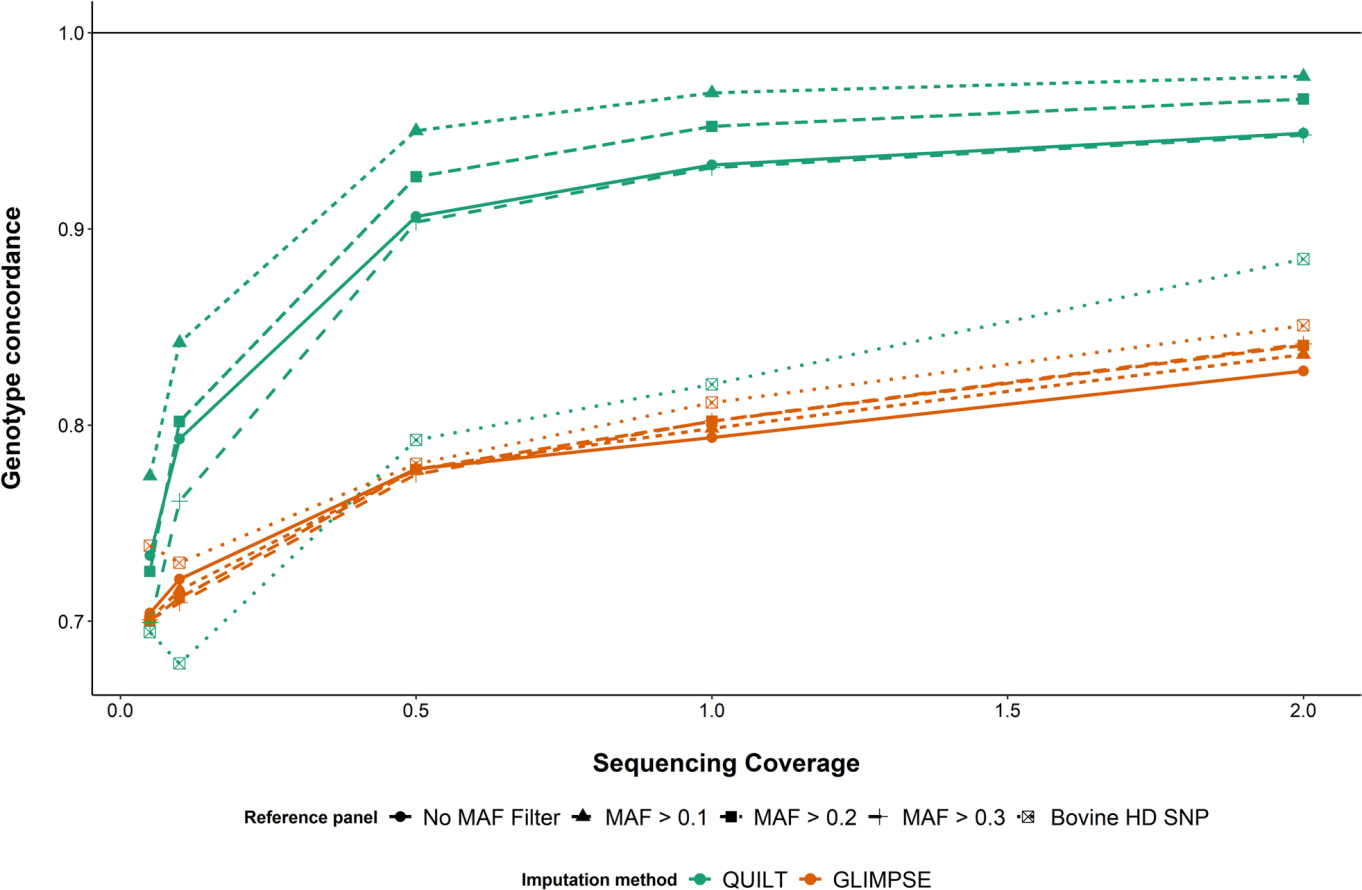
Genotype concordance for genotypes derived from low-coverage Oxford Nanopore Technologies (ONT) sequence data imputed using GLIMPSE and QUILT. Twelve animals were sequenced twice at two separate time points and genotypes were calculated separately for each of the sequencing runs. Five different sequencing coverages were evaluated for the two different imputation methods as well as five different imputation reference panel sizes. The largest imputation reference panel used 48 million SNP, a minor allele frequency (MAF) cutoff of 0.1, 0.2 and 0.3, was used to subset the 48 million SNP reference panel down. The final imputation reference panel used only the 700 k SNP in the bovine HD SNP array

The final question we looked to answer was, how reproducible are ONT-derived genotypes across sequencing runs? The twelve animals which were sequenced twice at two different time points in the study by Hayes et al. [27] were used to evaluate the reproducibility of low-coverage ONT genotyping using QUILT and GLIMPSE for imputation. The genotype concordance for each animal was calculated for all sites on the bovine HD SNP array, across the five different sequencing coverages and five different imputation SNP reference panels. The mean of the twelve animals was then taken as the true genotype concordance (Fig. 9). The genotype concordance using QUILT was higher than the concordance using GLIMPSE for all sequencing coverages and imputation reference panel combinations, except when the smallest imputation reference panel was used at 0.05 × sequencing coverage. The genotype concordance was highest for all sequencing coverages using the combination of QUILT with the second largest imputation reference panel (MAF > 0.1). At 2 × sequencing coverage, the genotype concordance for this combination was 0.98, this decreased gradually to 0.95 at 0.5 × sequencing coverage. The concordance decreased more significantly below 0.5 × to 0.84 and 0.77 at 0.1 × and 0.05 × sequencing coverage, respectively.

## Discussion

Near real-time, portable genomic prediction has a number of applications in agriculture and healthcare. Here, we have demonstrated in cattle that when combined with an appropriate imputation strategy skim-GBS using portable ONT sequencing is a suitable method for genomic prediction, with coverages as low as 0.1 × . Using the imputation packages QUILT and GLIMPSE, GEBV correlations were higher than or equal to correlations from low-density SNP arrays for sequencing coverages as low as 0.1 × and, at this coverage, the performance of animals relative to each other could accurately be separated into quartiles. We have also demonstrated that using large SNP reference panels with many more SNP than used in the genomic prediction equation significantly increases the accuracy of genomic prediction from low-coverage GBS. Overall, QUILT [21] in combination with large imputation reference panels outperformed the alternative genotyping and imputation methods across all accuracy metrics: imputation accuracy, genotype concordance, GEBV correlations and GEBV regression coefficients.

While a number of genotype specific accuracy metrics are available to compare imputation strategies, this study focused primarily on the accuracy of genomic prediction from the imputed genotypes. This approach was chosen as the study primarily aimed to investigate the optimum imputation strategy for the purpose of in situ genomic prediction. Imputation specific metrics such as genotype accuracy and concordance were used as a secondary means to evaluate the differences between QUILT and GLIMPSE, as they performed similarly based on genomic prediction metrics alone. The relative performance of the different strategies with regard to GEBV correlation and bias, highlighted the robustness of genomic prediction using a GBLUP model to a modest degree of imputation errors. This is in line with other literature that has reported little to no significant decrease in GEBV accuracies when imputation errors are introduced by decreasing SNP panel density from 50 k to as low as 3 k [35]. Nonetheless, irrespective of the accuracy metric used these results demonstrate skim-GBS using ONT data can objectively outperform low-density SNP arrays on both metrics when an appropriate imputation strategy is employed.

Calculating the prediction bias in the ONT predictions revealed a general trend of underpredicting GEBVs for CL score, BCS and HH, while over-predicting GEBVs for BW. This pattern was seen across all of the methods; however, QUILT still had the least prediction bias of the four methods across all coverages. A possible explanation for the trends in prediction bias observed could be systematic genotyping errors in SNP loci with a large phenotypic effect. For example, a SNP in the genomic region encoding *PLAG1* on chromosome 14 has previously been identified to have a significant effect on heifer fertility in Australian Brahman cattle [36], and if a systematic error were to occur for this region, predictions would likely be biased. Therefore, the presence of systematic errors in the QUILT imputed genotypes should be investigated and their subsequent proximity to SNP of large phenotypic effect determined.

Another possible explanation is that the bias is actually introduced by the process of imputing 35 k SNP array genotypes up to HD SNP array density. This implies that the prediction bias observed in body weight, for example, is actually caused by the low-density SNP array genotyping and not by the ONT GBS method. This is supported by the fact that when the ONT GEBVs are compared to the HD SNP array GEBVs, less prediction bias is observed with a more random spread, than when they are compared to the 35 k SNP array GEBVs. Furthermore, when comparing the 35 k SNP array GEBVs to the HD SNP array GEBVs a reasonable degree of bias was observed in body weight and hip height. This would suggest that some systematic genotype errors, which predominantly affect hip height and body weight GEBVs, are being introduced when imputing from the low-density 35 k array to the HD SNP array density. Further research should be carried out to verify if this is the case and investigate why the errors are being introduced.

QUILT’s ability to more accurately genotype and impute SNP from long-read sequence data is likely a result of the way in which it assigns haplotypes. Imputation packages that use VCF genotypes such as Beagle and GLIMPSE use SNP panels to construct known ancestral haplotypes in order to haplotype phase the genotypes. Missing loci are then imputed using the ancestral haplotypes. This approach is suitable for SNP array genotypes, where the genotypes themselves are independent observations. However, when using sequence data this approach ignores the fact that a single sequence read can span multiple SNP, meaning nearby SNP no longer need to be considered independent [21, 37]. QUILT uses the co-localization of nearby SNP within sequence reads to assign parental gametes and then imputes these two read groups (derived from each of the parental gametes) as haploids [21]. As SNP reference panel sizes increase this assumption becomes even more powerful because, on average, a single read spans more SNP. Given the average read length of 1792 bp for the ONT sequence data used for this study [31], reads on average spanned 30, 9, 6, 3 and 0.3 SNP for the five SNP reference panels, in order of largest to smallest, respectively. This likely meant that QUILT was able to more accurately assign SNP allele haplotypes, which ultimately increased the accuracy of genomic prediction. This implies that nearby SNP should not be treated as independent observations in future skim-GBS work. That is to say, when dealing with low-coverage sequence data (in particular long-read sequence data) genotyping and imputation approaches that use the co-localization of SNP on a sequence read to assign haplotypes should be used. We anticipate the popularity of such methods for genotyping and imputation of sequence data will increase as the method is further refined.

Although GLIMPSE was overall not as accurate as QUILT, it performed well even when used in combination with the smallest imputation reference panel (the HD SNP array) and was significantly more accurate than either Beagle method. This is likely a result of GLIMPSE using genotype likelihoods rather than hard-coded genotypes, such as the case with Beagle5.2. Genotype likelihoods allow for some of the individual read information to be maintained through to imputation when using GBS, however, are more computationally demanding to calculate and impute. Genotype likelihoods do not maintain haplotype of origin information which is available when a single read spans multiple SNP. This likely explains why GLIMPSE is relatively unaffected by decreasing imputation reference panel size. This means that in circumstances where very large imputation reference panels are available, QUILT is the better option as it can use the higher SNP density to accurately phase reads. When only small imputation reference panels are available, GLIMPSE may offer some increase in accuracy over QUILT. Overall, however, using QUILT with very large imputation reference panels was the most accurate for genomic prediction.

When comparing the accuracy of the QUILT and GLIMPSE imputed GEBVs to GEBVs derived from the bovine HD SNP array, there was no difference between 35 k SNP array GEBVs and ONT sequencing coverages as low as 0.1 × for QUILT and 0.5 × for GLIMPSE. This suggests for the purpose of genomic selection, 0.1 × sequencing coverage with ONT is as informative as low-medium density SNP arrays in beef cattle. Interestingly, no trend in prediction bias was observed when comparing the QUILT ONT GEBVs to the HD SNP array GEBVs. This suggests the underlying variation in GEBVs between the QUILT ONT and bovine HD SNP array predictions is random, unlike the variation in GEBVs between the ONT and 35 k array predictions. This means the long ONT sequence reads are potentially better able to account for decay in linkage disequilibrium (LD) than imputation from 35 k SNP array density up to HD array density. For example, at 0.1 × sequence coverage, theoretically, almost 5 million SNPs had sequence coverage from which to assign haplotypes using the whole genome SNP panel with no filter. Assuming random sampling of the haplotypes, we get 2.5 million SNPs for each parental haplotype, which is significantly more information than low-density SNP arrays for populations with greater LD decay. Examples of such populations include cattle breeds of *B. indicus* origin, such as Brahman and Droughtmaster, which were used in this study. This finding may suggest that low-coverage ONT genomic prediction may be particularly advantageous in mixed breed populations with a larger effective population size, such as Australia’s northern beef industry.

Previous studies have also found 0.5 × sequencing coverage suitable for skim-GBS for the purpose of genomic selection. Using Illumina sequencing data, Zhang et al. [38] reported high GEBV accuracies for coverages equal to or greater than 0.5 × in aquaculture species. While in the root crop cassava Long et al. [39] reported imputation accuracies from 0.5 × sequencing coverage were not different to accuracies from 5 × sequencing coverage using ONT data. By demonstrating 0.1 × sequencing coverage with ONT data is able to generate accurate GEBVs, we have further decreased the minimum sequencing coverage by five-fold, which has significant economic and practical implications for skim-GBS with ONT.

While the sequencing coverages for accurate imputation reported here are not as low as those reported for RR-GBS methods, the additional wet-lab procedures necessary for RR-GBS methods limit their portability. Additionally, given the low complexity of the genomes of most economically important livestock species, reduced-representation GBS methods are likely not necessary. For economically important species with highly complex genomes, such as wheat and sugar cane, reduced-representation GBS may still be the best option [10].

A potential solution to help handle complex genomes without the additional laboratory steps could be to use ONT’s adaptive sequencing technology [40] as a type of reduced-representation GBS method. Adaptive sequencing is an ONT-specific method to enrich or deplete desired genomic regions in real time. By enriching target SNP regions using adaptive sequencing, genome complexity could be decreased without the additional wet-lab steps of restriction enzyme digests. This method of ONT specific RR-GBS could decrease the required sequencing coverage for GBS on ONT platforms. This method could also be used to target QTL for monogenic traits alongside skim-GBS. Currently, limitations around computing power exist to make this method feasible outside of the lab. However, ONT’s partnership with NVIDIA could deliver the necessary improvements to make this method feasible at scale [41].

In this study, we compared ONT GEBVs to SNP array GEBVs using the same number of SNP markers for predictions. Future work should look to investigate the accuracy of ONT GEBVs against true breeding values and compare these results to SNP array GEBVs against the same true breeding values. Using this comparison, other methods to further increase the accuracy of ONT GEBVs could be better investigated. Such methods include using Bayesian-derived SNP effects to better estimate the distribution of effects, and incorporating more SNP into the prediction, rather than imputing to the whole genome sequence and then sub-sampling back to 641,000 SNPs. Both these methods have been shown to slightly increase prediction accuracies [42, 43] and would add little computational time once the SNP effects are calculated.

Reducing the size of the SNP reference panel had a significant effect on the imputation speed for all methods. Beagle5.2 was significantly faster than QUILT and GLIMPSE, which is a result of the increased computational complexity of using genotype likelihoods. Browning et al. [18] reported similar advantages in speed using Beagle5.0 over other imputation methods such as Minimac3, Minimac4 and Beagle4.1, particularly for large SNP panels. Although not as fast as Beagle5.2, QUILT offers a significant advantage in removing the need for prior genotyping. This decreases the complexity of file handling and depending on the genotyping approach used, decreases the overall turnaround time. Furthermore, QUILT was able to accurately impute from lower sequencing coverages than Beagle5.2 and GLIMPSE, meaning QUILT would further save time during data acquisition. Additionally, QUILT also allows pre-processed haplotype reference files to be loaded. This can be used to further decrease computational time where the same SNP reference panel is being used repeatedly. However, based on the imputation times reported here, data acquisition (i.e., sequencing time) will remain the rate-limiting step for real-time genomic prediction. Therefore, efforts to decrease the turnaround time should focus on decreasing the required sequencing coverage, not the speed of the bioinformatics pipeline.

In general, increasing the size of the reference panel increased imputation accuracy when using QUILT. However, this was not the case when increasing the panel size from the second largest panel (MAF > 0.1) to the largest reference panel (no MAF filter). Instead, a small decrease in imputation accuracy and GEBV correlations was observed across all coverages. It is believed this is related to the increased likelihood of sequencing errors appearing at rare variant loci, possibly causing non-reference SNP to be called at these loci and consequently introducing errors in the haplotype phasing and imputation. Given 33,202,930 rare SNP (MAF < 0.1) were removed from the unfiltered reference panel to create the second largest reference panel, it could be reasoned that up to 0.1% or 33,202 of those variants may overlap a sequencing error at 0.05 × , using a conservative ONT error rate of 2%. Therefore, it seems reasonable to assume sequencing errors may be the cause of the decrease in accuracy.

Despite, increasing the accuracy of genomic prediction and imputation accuracy, the removal of rare variants from the reference panels represents a limitation to the wider application of the imputation strategies evaluated here. For example, rare variants are useful for GWAS and genetic disease screening of some monogenic traits. Therefore, the filtered SNP panels in this study are only suitable for genomic prediction of polygenic traits. Incorporating rare disease loci, such as Pompes disease [44, 45] or horn/poll markers [45, 46] for cattle into the SNP panel would allow for screening of these dominant traits as well. However, it is unlikely accurate imputation of these genotypes will be possible at sequencing coverages below 1 × , due to the inverse correlation between imputation accuracy and MAF [39]. Additionally, the economic significance of traits such as Pompes disease and horn/poll would mean less error would be tolerated by producers. For instance, a bull incorrectly genotyped as homozygous poll (the desirable genotype) rather than heterozygous would mistakenly fetch a premium at a sale. Therefore, the ability for low coverage ONT data to accurately genotype rare causative variants must be validated. This is a potential area where targeted enrichment through adaptive sequencing or other methods could prove useful.

The accuracies achievable with QUILT at 0.1 × sequencing coverage suggest multiplexing of up to 40 human genome size samples on a single MinION (or up to 200 on a PromethION) flow cell could be possible. This would bring the reagent cost of this method down to below USD $40 per sample, while providing results in under 24 h [47]. With further optimisation of SNP panel design and the incorporation of new ONT technologies, the level of multiplexing possible will increase further still, making near real-time genomic prediction practically and economically feasible.

## Conclusions

Here we illustrated that genomic prediction can be accurately performed from ONT sequence data using sequencing coverages as low as 0.1 × with an appropriate imputation strategy. The resulting values are as accurate — if not more accurate — than low-density SNP array genotyping. This study illustrates that the computational requirements for in situ genomic prediction are available, and the sequencing coverages reported here make ONT genotyping economically viable ultimately making on-farm genotyping a possibility in the near future.

## Methods

### Ethics

Tail hair samples from 48 heifers spread across three properties were collected under the ethics approval number QAAFI/269/17. These heifers were Brahman and Droughtmaster breed animals, which are of predominantly *B. indicus* origin. An additional 14 tail hair samples were collected at a different time point from a subset of the original animals making a total of 62 samples.

### SNP array genotyping

All animals (*n* = 41) were genotyped on the 35 k GGP TropBeef SNP array (Neogen, Lansing, MI) and the genotypes were imputed up to the bovine HD array density using Fimpute [34] and a panel of 3140 cattle genotyped on the bovine HD SNP array (Illumina, San Diego, CA). A subset of 19 animals belonging to a single herd were also genotyped on the bovine HD SNP array (Illumina, San Diego, CA).

### DNA preparation and sequencing

The DNA preparation and sequencing methods described extensively in Lamb et al. [31] and Hayes et al. [27] were used to sequence all 64 samples. Briefly, genomic DNA was extracted from the tail hairs using the Gentra Pure gene Tissue Kit (Qiagen) and the ligation sequencing kit (SQK-LSK109; ONT, Oxford, United Kingdom) was used to prepare the sequencing library. Libraries were loaded onto MinION flow cells (R9.4.1; ONT, Oxford, UK) and run for up to 96 h with at least three nuclease flushes and reloading.

### Genotype calling

Guppy (version 4.2.2; ONT, Oxford, UK) was used to base-call the raw fast5 files and the subsequent fastq files were subsampled down to 2 × , 1 × , 0.5 × , 0.1 × and 0.05 × for each animal. Each subsampled fastq was aligned to the *Bos taurus* reference genome ARS-UCD version 1.2 [28] using Minimap2 (version 2.20) [29] and the default ONT mapping settings. Samtools (version 1.14) [48] was used to create an mpileup and two different genotyping methods were used to assign genotypes for downstream imputation. The first method used a variable minimum allele count to genotype loci based on read depth as described in Lamb et al. [31]. This method grouped loci with similar coverage and called genotypes using a minimum allele count for each coverage bin. The second method used the original genotype likelihood method implemented in GATK [32] to calculate genotype likelihoods (GL) for imputation:

$$GL= \prod_{i=1}^{M}\mathrm{Pr}\left({b}_{i}|G=\left\{{A}_{1}, {A}_{2}\right\}\right)= \prod_{i=1}^{M}(\frac{1}{2}\mathrm{Pr}\left({b}_{i}|{A}_{1}\right))+ (\frac{1}{2}\mathrm{Pr}\left({b}_{i}|{A}_{2}\right))$$

$$\mathrm{Pr}(b|A)=\left\{\begin{array}{c}\frac{e}{3} :b \ne A\\ 1-e :b=A\end{array}\right.$$

where *G* is the true genotype, *M* is the sequencing depth, *e* is the probability of an error calculated using the phred scaled qscore and ${b}_{i}$ is the observed base in read *i*.

This second method also accounted for methylation errors in ONT sequence data by only genotyping potential methylation sites using the non-methylated strand. For example, to genotype a cytosine flanked by a guanine on the forward strand, only reads on the reverse strand were considered.

### Imputation

Two imputation packages, QUILT (version 1.0.1) [21] and Beagle (version 4.1 and 5.2) [18] were used to impute missing genotypes. A SNP panel of 48,203,338 million SNPs and 1,208 animals comprised of breeds found in northern Australia was used as the reference. The 48 million SNPs were a subset of SNPs from the 1000 Bull Genomes project [30], known to segregate in the northern Australian beef herd. Three MAF filters (MAF > 0.1, MAF > 0.2 and MAF > 0.3) were further used to reduce the size of the SNP panel (while retaining the bovine HD SNP) and examine the effect of smaller SNP panels on the speed and accuracy of imputation. The imputation time for each animal and combination of SNP panel size, coverage and imputation method was recorded.

### SNP-BLUP method for EBVs

Genomic estimated breeding values $(\mathrm{\widehat{a}})$ for four traits: BW, HH, BCS and CL scores were then calculated using 641,163 SNP loci with SNP effects from Hayes et al. [49] as described in Lamb et al. [31] using:

$$\hat{a} =M\widehat{g}$$

where the 35 k SNP array genotypes were contained in an n by m matrix M, where n was the number of animals and m was the number of SNP loci (641,163) and $\widehat{g}$ was a vector of predicted SNP effects with length m.

The estimated phenotypes from the imputed ONT genotypes ($\widehat{b}$) were then calculated using the same equation but replacing the 35 k SNP array genotypes in $\widehat{g}$ with the imputed ONT genotypes. Finally, estimated phenotypes for the 19 animals with 728 k SNP array genotypes ($\widehat{c})$ were then also calculated by once again replacing the genotypes in $\widehat{g}$ with genotypes from the 728 k SNP array corresponding to the same 641 k loci.

The accuracy of the ONT GEBVs was then compared using linear models to calculate the correlation between the 35 k SNP array GEBVs and ONT GEBVs $(\widehat{a} \sim \widehat{b})$. The regression of $\mathrm{\widehat{a} } \sim \widehat{b}$ was also used to calculate the prediction bias which was defined here as the regression coefficient of $\hat{a} \sim \widehat{b}$ minus 1. The correlation between the bovine HD SNP array GEBV and ONT GEBV imputed using QUILT was also calculated $\widehat{(c} \sim {\widehat{b}}_{QUILT})$ and compared to the correlation between $\widehat{c}$ and $\mathrm{\hat{a} }$.

Samples with coverage of more than one standard deviation from the mean sequence coverage for each depth were dropped from the analysis. For example, at 2 × coverage seven samples had a sequencing depth of less than one standard deviation (0.37) from the mean of 1.79 × and were therefore dropped from the analysis for this coverage. The actual sequencing depth was calculated as bases mapped to the reference genome while the original read subsampling for each coverage group was done according to the raw fastq files. This therefore accounts for the difference between actual sequencing depth and coverage group and provides a more accurate estimate of GEBV accuracy for a given coverage calculated in situ.

### Genotype concordance and imputation accuracy

The genotype concordance between the 14 duplicate animals was calculated at the HD SNP array loci using the package Bcftools. For each duplicate sample, the vcf files were firstly filtered to remove SNP not on the HD SNP array and then compared using the bcftools stats function from Bcftools. Overall genotype concordance was recorded for each sample.

The imputation accuracy for the imputed ONT genotypes and imputed 35 k genotypes was calculated by comparing the imputed genotypes to the available HD SNP array genotypes at the same loci used for the genomic prediction. Imputation accuracy was defined as the total number of matching genotypes divided by 641,163 (the number of SNP used for genomic prediction).

### Supplementary Information

**Additional file 1: Table S1. **Summary statistics of the ONT sequencing data for each sample used.** Table S2.** Breed composition breakdown for the SNP reference panel used for imputation. **Figure S1.** Principal component plot for a subset of the 1,208 animals in the SNP reference panel and 62 animals sequenced using Oxford Nanopore Technologies’ (ONT) MinION as part of this study. **Figure 2.** Correlations between hip height (HH) genomic estimated breeding values (GEBV) derived from 35k SNP array genotypes and HH GEBVs derived from Oxford Nanopore Technologies (ONT) data. ONT GEBVs were imputed using four different imputation strategies and across five sequencing coverages. SNP reference panel size is indicated by the minor allele frequency (MAF) filter on the right-hand side in descending order of size from top to bottom. The largest panel had 48,203,338 SNP and was referred to as the No MAF filter panel, while the smallest panel was referred to as the bovine high density (HD) SNP which had only the 641k SNP used to calculate the GEBVs. Error bars indicate 95% confidence of the Pearson correlation. **Figure S3.** Correlations between *corpus luteum *score (CL score) genomic estimated breeding values (GEBV) derived from 35k SNP array genotypes and HH GEBVs derived from Oxford Nanopore Technologies (ONT) data. ONT GEBVs were imputed using four different imputation strategies and across five sequencing coverages in descending order of size from top to bottom. The largest panel had 48,203,338 SNP and was referred to as the No MAF filter panel, while the smallest panel was referred to as the bovine high density (HD) SNP which had only the 641k SNP used to calculate the GEBVs. SNP reference panel size is indicated by the minor allele frequency (MAF) filter on the right-hand side. Error bars indicate 95% confidence in the Pearson correlation. **Figure S4.** Correlations between body condition score (BCS) genomic estimated breeding values (GEBV) derived from 35k SNP array genotypes and HH GEBVs derived from Oxford Nanopore Technologies (ONT) data. ONT GEBVs were imputed using four different imputation strategies and across five sequencing coverages. SNP reference panel size is indicated by the minor allele frequency (MAF) filter on the right-hand side in descending order of size from top to bottom. The largest panel had 48,203,338 SNP and was referred to as the No MAF filter panel, while the smallest panel was referred to as the bovine high density (HD) SNP which had only the 641k SNP used to calculate the GEBVs. Error bars indicate 95% confidence in the Pearson correlation.** Figure S5.** A) Correlations between genomic estimated breeding values (GEBVs) derived from Oxford Nanopore Technologies (ONT) sequence data and GEBVs derived from bovine HD SNP array genotypes for hip height (HH). ONT derived GEBVs were imputed using QUILT and GLIMPSE and calculated across five coverages and five SNP panels. The different SNP reference panels were created using minor allele frequency (MAF) filters to reduce the size of the panels down from whole genome sequence SNP. The largest panel had 48,203,338 SNP and was referred to as the No MAF filter panel, while the smallest panel was referred to as the bovine high definition (HD) SNP panel and featured only the 641k SNP used to calculate the GEBVs. SNP array genotypes were from the Illumina bovine HD SNP array. The correlation for each trait between GEBVs calculated from the 35k GGP SNP array imputed to 700k and GEBVs calculated from the Illumina bovine HD SNP array are indicated by the dashed line. The colour of each bar indicates how well the ONT derived GEBV accuracies compare to the 35K SNP array accuracies. Error bars indicate 95% confidence interval of the Pearson correlation. B) Genomic prediction bias for body weight (BW), defined as> ${\mathrm\beta}_2-1$ >, where ${\mathrm\beta}_2$ is the regression coefficient of the bovine HD SNP array genomic estimated breeding value (GEBV) ~ Oxford Nanopore Technologies GEBV derived using QUILT and GLIMPSE. The prediction bias of the HD SNP array GEBVs ~ 35k SNP array GEBVs are displayed for each trait by the dotted lines, where the colour of the line corresponds to the colour of the trait in the figure legend. **Figure S6.** A) Correlations between genomic estimated breeding values (GEBVs) derived from Oxford Nanopore Technologies (ONT) sequence data and GEBVs derived from bovine HD SNP array genotypes for *corpus luteum *score (CL score). ONT derived GEBVs were imputed using QUILT and GLIMPSE and calculated across five coverages and five SNP panels. The different SNP reference panels were created using minor allele frequency (MAF) filters to reduce the size of the panels down from whole genome sequence SNP. The largest panel had 48,203,338 SNP and was referred to as the No MAF filter panel, while the smallest panel was referred to as the bovine high definition (HD) SNP panel and featured only the 641k SNP used to calculate the GEBVs. SNP array genotypes were from the Illumina bovine HD SNP array. The correlation for each trait between GEBVs calculated from the 35k GGP SNP array imputed to 700k and GEBVs calculated from the Illumina bovine HD SNP array are indicated by the dashed line. The colour of each bar indicates how well the ONT derived GEBV accuracies compare to the 35K SNP array accuracies. Error bars indicate 95% confidence interval of the Pearson correlation. B) Genomic prediction bias for body weight (BW), defined as ${\mathrm\beta}_2-1$, where ${\mathrm\beta}_2$ [endif][endif] is the regression coefficient of the bovine HD SNP array genomic estimated breeding value (GEBV) ~ Oxford Nanopore Technologies GEBV derived using QUILT and GLIMPSE. The prediction bias of the HD SNP array GEBVs ~ 35k SNP array GEBVs are displayed for each trait by the dotted lines, where the colour of the line corresponds to the colour of the trait in the figure legend. **Figure S7. **A) Correlations between genomic estimated breeding values (GEBVs) derived from Oxford Nanopore Technologies (ONT) sequence data and GEBVs derived from bovine HD SNP array genotypes for body condition score (BCS). ONT derived GEBVs were imputed using QUILT and GLIMPSE and calculated across five coverages and five SNP panels. The different SNP reference panels were created using minor allele frequency (MAF) filters to reduce the size of the panels down from whole genome sequence SNP. The largest panel had 48,203,338 SNP and was referred to as the No MAF filter panel, while the smallest panel was referred to as the bovine high definition (HD) SNP panel and featured only the 641k SNP used to calculate the GEBVs. SNP array genotypes were from the Illumina bovine HD SNP array. The correlation for each trait between GEBVs calculated from the 35k GGP SNP array imputed to 700k and GEBVs calculated from the Illumina bovine HD SNP array are indicated by the dashed line. The colour of each bar indicates how well the ONT derived GEBV accuracies compare to the 35K SNP array accuracies. Error bars indicate 95% confidence interval of the Pearson correlation. B) Genomic prediction bias for body weight (BW), defined as ${\mathrm\beta}_2-1$  , where ${\mathrm\beta}_2$ [endif][endif] is the regression coefficient of the bovine HD SNP array genomic estimated breeding value (GEBV) ~ Oxford Nanopore Technologies GEBV derived using QUILT and GLIMPSE. The prediction bias of the HD SNP array GEBVs ~ 35k SNP array GEBVs are displayed for each trait by the dotted lines, where the colour of the line corresponds to the colour of the trait in the figure legend.

## Data Availability

The datasets generated and/or analysed during the current study are available in the NCBI SRA BioProject repository [50].

## Abbreviations

BCS: Body condition score
BW: Body weight
CL score: *Corpus luteum* Score
GBS: Genotyping-by-sequencing
GEBV: Genomic estimated breeding values
HD: High density
HH: Hip height
HH: Hip height
LD: Low density
MAC: Minimum allele count
MAF: Minor allele frequency
ONT: Oxford Nanopore Technologies
QTL: Quantitative trait loci
RR-GBS: Reduced representation genotyping-by-sequencing
Skim-GBS: Skim genotyping-by-sequencing
SNP: Single nucleotide polymorphism
