# Supplementary material for: Imputation strategies for genomic prediction using nanopore sequencing
Source: BMC Biol. 2023 Dec 8;21:286. doi: 10.1186/s12915-023-01782-0 (PMC10709982; doi:10.1186/s12915-023-01782-0)
Supplement: Supplementary file 1 — Additional file 1: Table S1. Summary statistics of the ONT sequencing data for each sample used. Table S2. Breed composition breakdown for the SNP reference panel used for imputation. Figure S1. Principal component plot for a subset of the 1,208 animals in the SNP reference panel and 62 animals sequenced using Oxford Nanopore Technologies’ (ONT) MinION as part of this study. Figure 2. Correlations between hip height (HH) genomic estimated breeding values (GEBV) derived from 35k SNP array genotypes and HH GEBVs derived from Oxford Nanopore Technologies (ONT) data. ONT GEBVs were imputed using four different imputation strategies and across five sequencing coverages. SNP reference panel size is indicated by the minor allele frequency (MAF) filter on the right-hand side in descending order of size from top to bottom. The largest panel had 48,203,338 SNP and was referred to as the No MAF filter panel, while the smallest panel was referred to as the bovine high density (HD) SNP which had only the 641k SNP used to calculate the GEBVs. Error bars indicate 95% confidence of the Pearson correlation. Figure S3. Correlations between corpus luteum score (CL score) genomic estimated breeding values (GEBV) derived from 35k SNP array genotypes and HH GEBVs derived from Oxford Nanopore Technologies (ONT) data. ONT GEBVs were imputed using four different imputation strategies and across five sequencing coverages in descending order of size from top to bottom. The largest panel had 48,203,338 SNP and was referred to as the No MAF filter panel, while the smallest panel was referred to as the bovine high density (HD) SNP which had only the 641k SNP used to calculate the GEBVs. SNP reference panel size is indicated by the minor allele frequency (MAF) filter on the right-hand side. Error bars indicate 95% confidence in the Pearson correlation. Figure S4. Correlations between body condition score (BCS) genomic estimated breeding values (GEBV) derived from 35k SNP array genotypes and HH GE [file 12915_2023_1782_MOESM1_ESM.docx]

**Supplementary Tables and Figures**

**Supplementary Table 1: Summary statistics of the ONT sequencing data for each sample used.**

| **Sample ID** | **Bases mapped (Mbp)** | | | | | **Total bases produced from flow cell (Gbp)** | **Average read length (Kbp)** | **Average read quality (Phred score)** | **Mapping Percent** |
| --- | --- | --- | --- | --- | --- | --- | --- | --- | --- |
|  | **0.05x** | **0.1x** | **0.5x** | **1x** | **2x** |  |  |  |  |
| 1 | 0.14 | 0.28 | 1.42 | 2.85 | 5.64 | 25.87 | 1.44 | 20.50 | 0.88 |
| 2 | 0.14 | 0.29 | 1.43 | 2.88 | 5.70 | 21.95 | 3.35 | 20.20 | 0.89 |
| 3 | 0.15 | 0.29 | 1.45 | 2.91 | 5.78 | 19.74 | 3.42 | 20.70 | 0.90 |
| 4 | 0.15 | 0.29 | 1.47 | 2.93 | 5.82 | 15.98 | 3.85 | 20.80 | 0.92 |
| 5 | 0.15 | 0.29 | 1.46 | 2.92 | 5.77 | 21.15 | 3.29 | 20.90 | 0.92 |
| 6 | 0.14 | 0.28 | 1.39 | 2.78 | 5.51 | 26.00 | 1.77 | 20.20 | 0.86 |
| 7 | 0.15 | 0.30 | 1.51 | 2.80 | 2.80 | 8.97 | 1.25 | 22.50 | 0.97 |
| 8 | 0.14 | 0.28 | 1.41 | 2.83 | 5.61 | 8.15 | 2.45 | 20.00 | 0.89 |
| 9 | 0.14 | 0.29 | 1.44 | 2.88 | 5.71 | 11.38 | 1.63 | 21.00 | 0.90 |
| 10 | 0.14 | 0.28 | 1.41 | 2.82 | 5.59 | 17.18 | 1.27 | 20.10 | 0.88 |
| 11 | 0.14 | 0.29 | 1.44 | 2.88 | 5.72 | 14.33 | 2.72 | 21.10 | 0.90 |
| 12 | 0.14 | 0.28 | 1.42 | 2.84 | 5.63 | 28.22 | 2.10 | 19.70 | 0.89 |
| 13 | 0.14 | 0.29 | 1.46 | 2.92 | 5.79 | 20.82 | 3.74 | 20.80 | 0.92 |
| 14 | 0.14 | 0.29 | 1.45 | 2.91 | 5.77 | 17.00 | 2.11 | 20.80 | 0.91 |
| 15 | 0.14 | 0.29 | 1.44 | 2.88 | 5.71 | 25.01 | 3.93 | 20.80 | 0.90 |
| 16 | 0.14 | 0.28 | 1.41 | 2.52 | 2.52 | 4.00 | 1.09 | 20.00 | 0.88 |
| 17 | 0.14 | 0.28 | 1.41 | 2.82 | 3.41 | 4.75 | 2.03 | 20.30 | 0.87 |
| 18 | 0.14 | 0.29 | 1.43 | 2.86 | 5.67 | 17.33 | 1.62 | 20.30 | 0.89 |
| 19 | 0.15 | 0.30 | 1.49 | 2.99 | 5.93 | 29.04 | 2.45 | 21.20 | 0.95 |
| 20 | 0.15 | 0.29 | 1.47 | 2.94 | 5.83 | 20.70 | 2.41 | 20.50 | 0.94 |
| 21 | 0.14 | 0.29 | 1.44 | 2.88 | 5.71 | 16.64 | 7.34 | 19.80 | 0.87 |
| 21_rep | 0.14 | 0.29 | 1.45 | 2.90 | 5.75 | 20.69 | 3.12 | 20.60 | 0.91 |
| 23 | 0.15 | 0.29 | 1.47 | 2.93 | 5.83 | 13.57 | 3.99 | 21.10 | 0.93 |
| 24 | 0.14 | 0.29 | 1.45 | 2.89 | 5.74 | 19.22 | 6.60 | 20.10 | 0.91 |
| 25 | 0.14 | 0.29 | 1.45 | 2.90 | 5.76 | 16.93 | 4.05 | 20.50 | 0.92 |
| 26 | 0.14 | 0.29 | 1.44 | 2.88 | 5.71 | 8.10 | 3.16 | 20.00 | 0.90 |
| 26_rep | 0.15 | 0.30 | 1.47 | 2.95 | 5.85 | 19.24 | 4.57 | 20.90 | 0.94 |
| 28 | 0.15 | 0.29 | 1.47 | 2.94 | 5.83 | 13.00 | 4.57 | 21.50 | 0.94 |
| 29 | 0.14 | 0.29 | 1.44 | 2.89 | 5.73 | 10.26 | 2.57 | 20.60 | 0.90 |
| 30 | 0.15 | 0.29 | 1.46 | 2.92 | 3.27 | 19.41 | 5.25 | 21.30 | 0.93 |
| 30_rep | 0.15 | 0.30 | 1.51 | 3.01 | 5.97 | 17.47 | 4.83 | 22.50 | 0.97 |
| 32 | 0.14 | 0.28 | 1.42 | 2.83 | 5.62 | 13.71 | 3.47 | 20.20 | 0.87 |
| 32_rep | 0.14 | 0.29 | 1.43 | 2.86 | 5.68 | 11.08 | 3.47 | 18.80 | 0.90 |
| 34 | 0.15 | 0.29 | 1.46 | 2.92 | 5.79 | 23.69 | 3.88 | 21.20 | 0.90 |
| 35 | 0.14 | 0.28 | 1.41 | 2.83 | 5.60 | 14.90 | 3.51 | 18.20 | 0.85 |
| 35_rep | 0.15 | 0.29 | 1.46 | 2.91 | 5.77 | 18.59 | 3.54 | 20.90 | 0.91 |
| 37 | 0.14 | 0.29 | 1.45 | 2.91 | 5.76 | 34.96 | 2.94 | 20.90 | 0.92 |
| 38 | 0.14 | 0.28 | 1.42 | 2.85 | 5.49 | 7.96 | 2.70 | 19.80 | 0.90 |
| 38_rep | 0.14 | 0.29 | 1.44 | 2.87 | 5.70 | 34.91 | 3.13 | 20.20 | 0.90 |
| 40 | 0.14 | 0.28 | 1.42 | 2.84 | 5.63 | 11.99 | 2.51 | 19.20 | 0.89 |
| 40_rep | 0.14 | 0.29 | 1.43 | 2.86 | 5.67 | 9.43 | 2.93 | 18.70 | 0.89 |
| 42 | 0.15 | 0.29 | 1.45 | 2.89 | 5.75 | 30.78 | 5.29 | 20.40 | 0.90 |
| 43 | 0.15 | 0.29 | 1.44 | 2.89 | 5.73 | 8.13 | 2.69 | 19.80 | 0.91 |
| 43_rep | 0.14 | 0.29 | 1.45 | 2.91 | 5.79 | 13.35 | 3.53 | 19.80 | 0.92 |
| 45 | 0.15 | 0.30 | 1.49 | 2.98 | 5.00 | 7.40 | 2.13 | 21.00 | 0.95 |
| 45_rep | 0.14 | 0.29 | 1.44 | 2.88 | 5.70 | 22.67 | 3.13 | 19.50 | 0.90 |
| 47 | 0.14 | 0.29 | 1.45 | 2.90 | 5.75 | 16.71 | 3.02 | 20.20 | 0.91 |
| 47_rep | 0.15 | 0.30 | 1.50 | 3.00 | 5.94 | 34.25 | 4.18 | 21.80 | 0.96 |
| 49 | 0.15 | 0.30 | 1.52 | 3.04 | 6.03 | 41.11 | 3.27 | 21.70 | 0.99 |
| 49_rep | 0.15 | 0.30 | 1.50 | 2.83 | 5.95 | 34.77 | 2.95 | 20.90 | 0.96 |
| 51 | 0.15 | 0.30 | 1.49 | 2.97 | 5.89 | 13.39 | 1.54 | 21.10 | 0.94 |
| 51_rep | 0.15 | 0.30 | 1.51 | 2.81 | 4.17 | 34.85 | 4.09 | 21.20 | 0.97 |
| 53 | 0.14 | 0.28 | 1.41 | 2.81 | 5.58 | 11.35 | 2.71 | 18.60 | 0.88 |
| 53_rep | 0.14 | 0.29 | 1.45 | 2.90 | 5.76 | 33.06 | 4.82 | 19.90 | 0.91 |
| 55 | 0.14 | 0.28 | 1.42 | 2.85 | 5.66 | 9.79 | 2.18 | 19.30 | 0.89 |
| 55_rep | 0.14 | 0.29 | 1.45 | 2.90 | 5.75 | 19.53 | 5.41 | 20.00 | 0.91 |
| 57 | 0.15 | 0.29 | 1.45 | 2.90 | 5.75 | 24.83 | 2.96 | 20.70 | 0.91 |
| 58 | 0.15 | 0.29 | 1.45 | 2.91 | 5.79 | 17.35 | 4.19 | 20.70 | 0.92 |
| 59 | 0.14 | 0.29 | 1.43 | 2.87 | 5.69 | 21.54 | 2.08 | 20.30 | 0.90 |
| 60 | 0.15 | 0.30 | 1.52 | 3.03 | 6.01 | 27.09 | 2.51 | 22.30 | 0.97 |
| 61 | 0.14 | 0.29 | 1.44 | 2.87 | 5.68 | 30.75 | 1.75 | 20.20 | 0.90 |
| 62 | 0.15 | 0.30 | 1.52 | 3.03 | 6.01 | 27.40 | 2.14 | 21.60 | 0.98 |
|  |  |  |  |  |  |  |  |  |  |
|  |  |  |  |  |  |  |  |  |  |

| **Breed** | ***n*** |
| --- | --- |
| Angus | 212 |
| Africander | 5 |
| Beefmaster | 10 |
| Boran | 11 |
| Brahman | 144 |
| Brangus | 49 |
| British shorthorn | 23 |
| Charolais | 83 |
| Droughtmaster | 75 |
| Hereford | 74 |
| Limousin | 60 |
| Murray Grey | 2 |
| Red Danish Cattle | 26 |
| Santa Getrudis | 24 |
| Sahiwal | 28 |
| Senepol | 12 |
| Shorthorn | 1 |
| Simmental x Angus | 46 |
| Texas Longhorn | 2 |
| Uganda Admixed | 260 |
| Tropical Composite | 61 |
| **Total** | **1208** |

**Supplementary Table 2: Breed composition breakdown for the SNP reference panel used for imputation.**


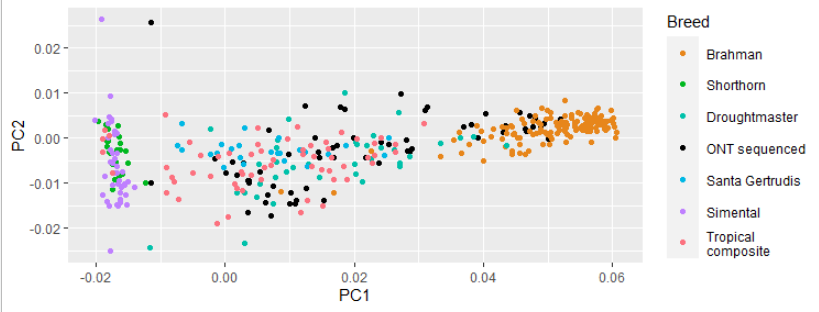


**Supplementary Figure 1. Principal component plot for a subset of the 1,208 animals in the SNP reference panel and 62 animals sequenced using Oxford Nanopore Technologies’ (ONT) MinION as part of this study.**

##
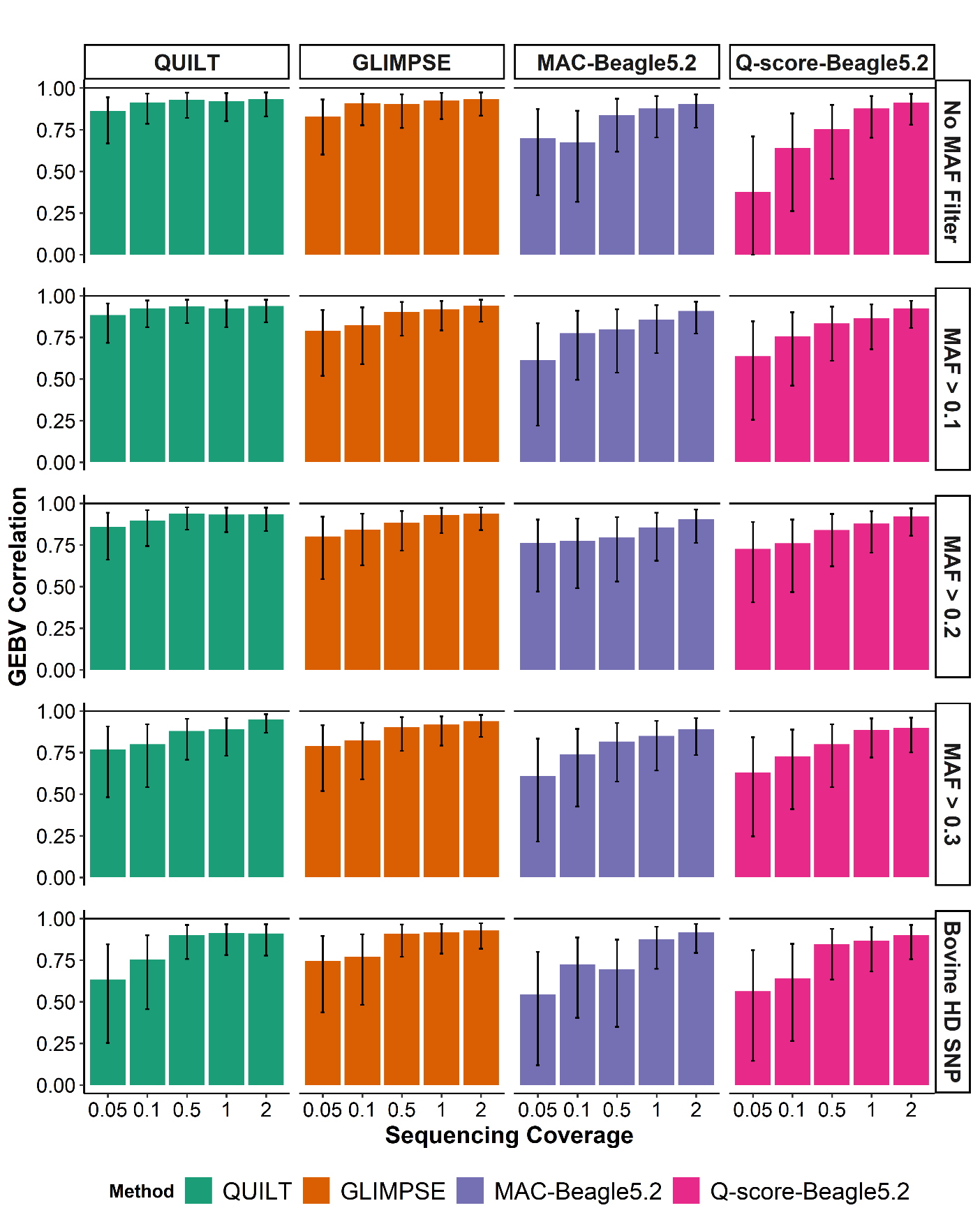


## **Supplementary Figure 2. Correlations between hip height (HH) genomic estimated breeding values (GEBV) derived from 35k SNP array genotypes and HH GEBVs derived from Oxford Nanopore Technologies (ONT) data. ONT GEBVs were imputed using four different imputation strategies and across five sequencing coverages. SNP reference panel size is indicated by the minor allele frequency (MAF) filter on the right-hand side in descending order of size from top to bottom. The largest panel had 48,203,338 SNP and was referred to as the No MAF filter panel, while the smallest panel was referred to as the bovine high density (HD) SNP which had only the 641k SNP used to calculate the GEBVs. Error bars indicate 95% confidence of the Pearson correlation.**


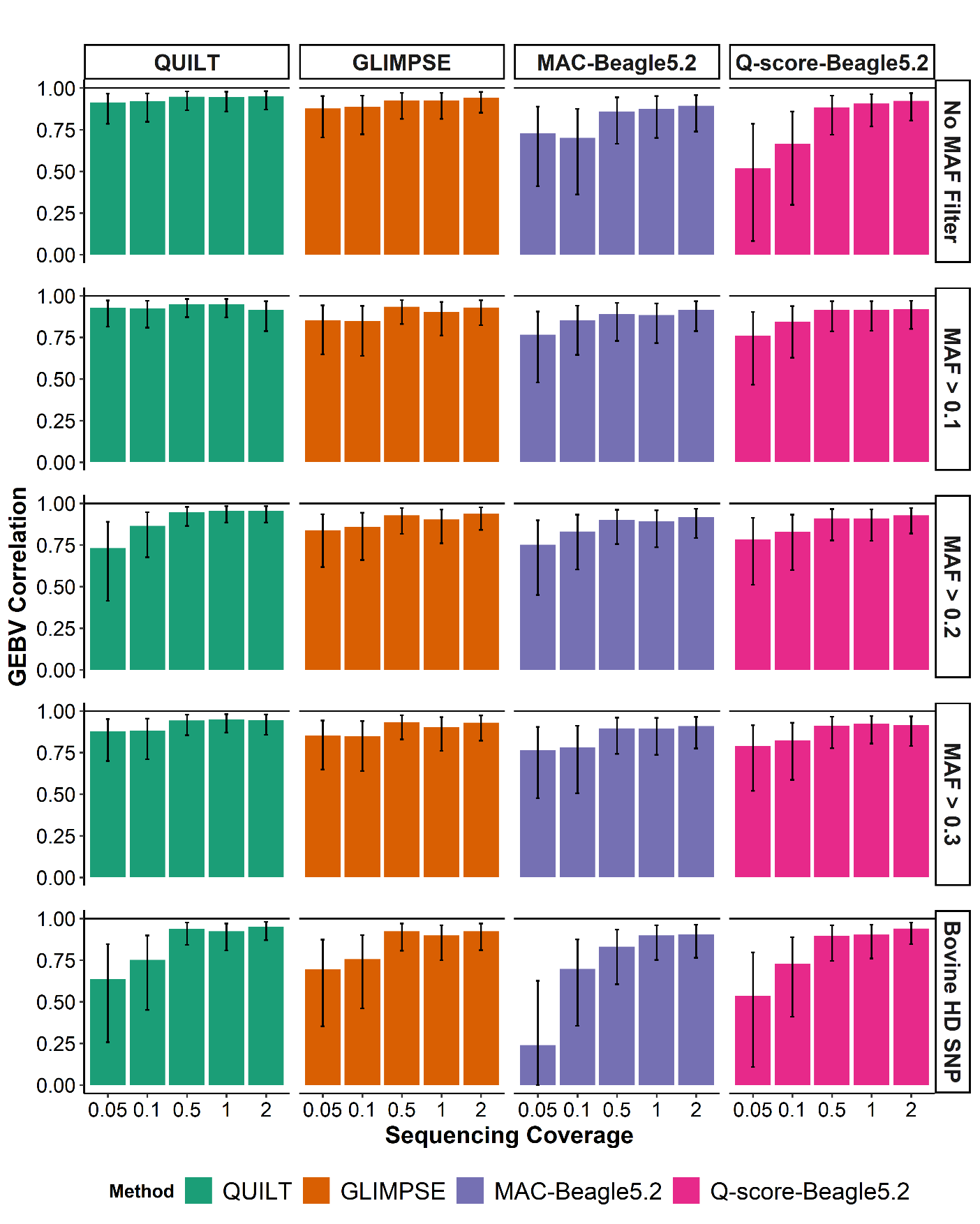


**Supplementary Figure 3. Correlations between *corpus luteum* score (CL score) genomic estimated breeding values (GEBV) derived from 35k SNP array genotypes and HH GEBVs derived from Oxford Nanopore Technologies (ONT) data. ONT GEBVs were imputed using four different imputation strategies and across five sequencing coverages in descending order of size from top to bottom. The largest panel had 48,203,338 SNP and was referred to as the No MAF filter panel, while the smallest panel was referred to as the bovine high density (HD) SNP which had only the 641k SNP used to calculate the GEBVs. SNP reference panel size is indicated by the minor allele frequency (MAF) filter on the right-hand side. Error bars indicate 95% confidence in the Pearson correlation.**


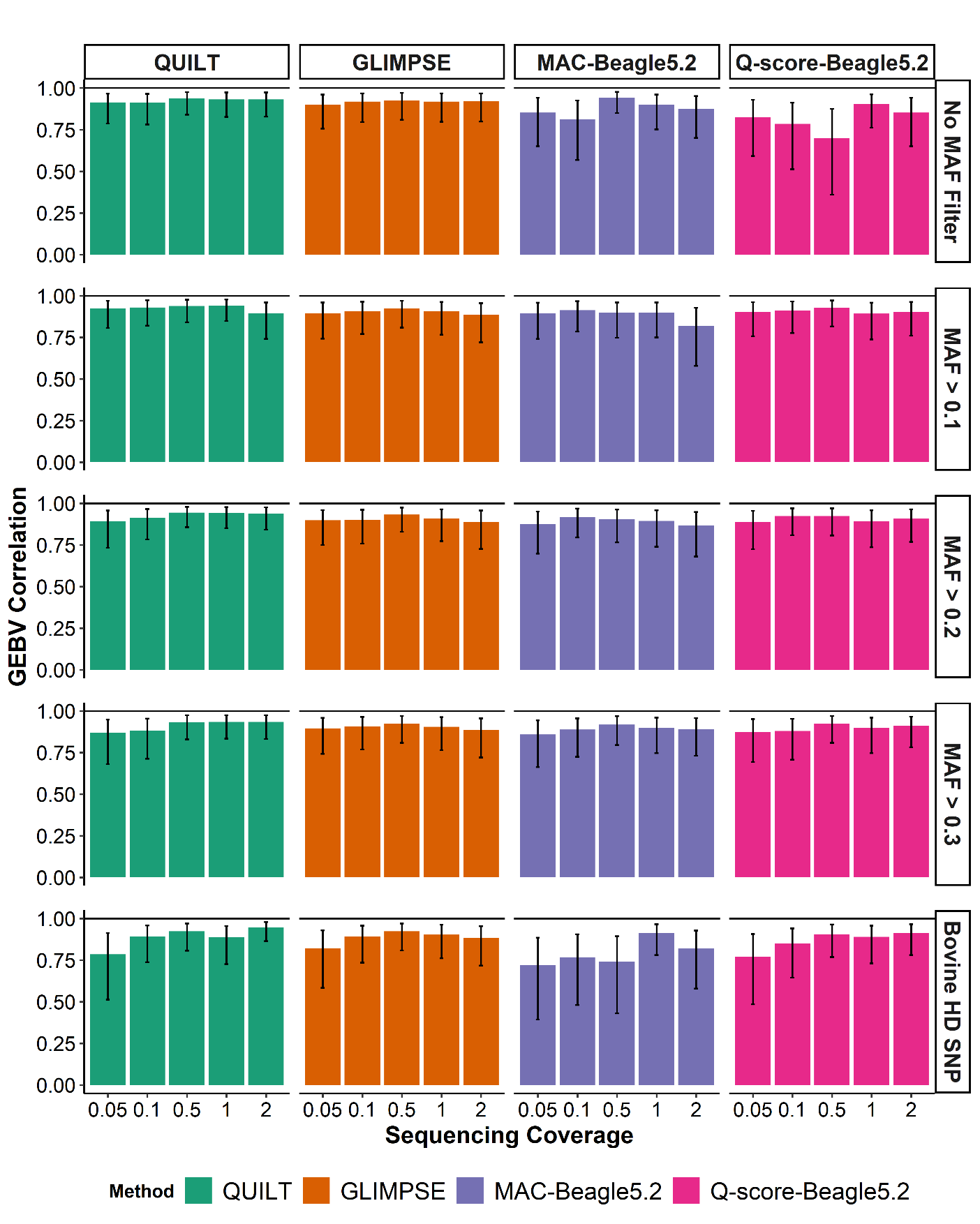


**Supplementary Figure 4. Correlations between body condition score (BCS) genomic estimated breeding values (GEBV) derived from 35k SNP array genotypes and HH GEBVs derived from Oxford Nanopore Technologies (ONT) data. ONT GEBVs were imputed using four different imputation strategies and across five sequencing coverages. SNP reference panel size is indicated by the minor allele frequency (MAF) filter on the right-hand side in descending order of size from top to bottom. The largest panel had 48,203,338 SNP and was referred to as the No MAF filter panel, while the smallest panel was referred to as the bovine high density (HD) SNP which had only the 641k SNP used to calculate the GEBVs. Error bars indicate 95% confidence in the Pearson correlation.**

**
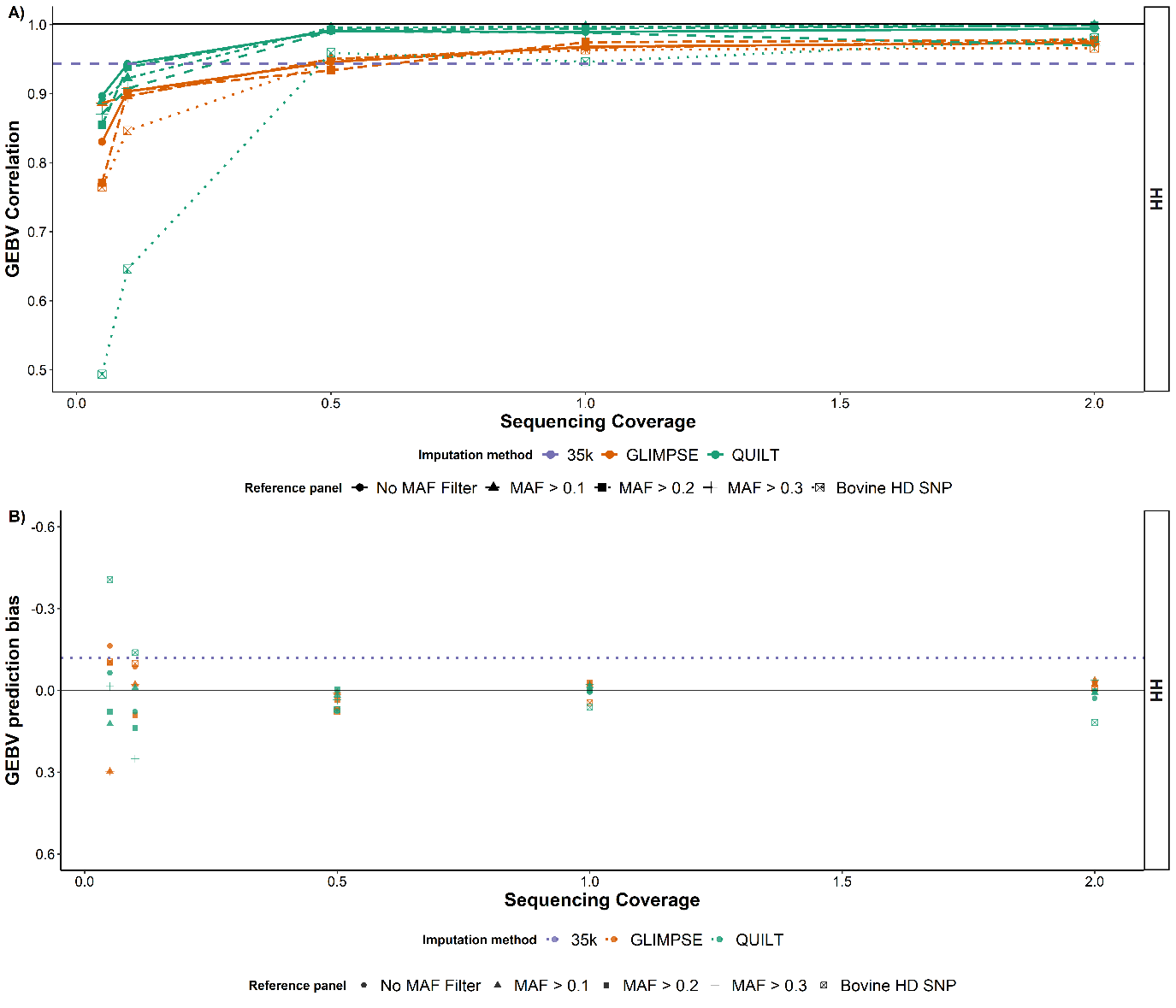
**

**Supplementary Figure 5. A)** **Correlations between genomic estimated breeding values (GEBVs) derived from Oxford Nanopore Technologies (ONT) sequence data and GEBVs derived from bovine HD SNP array genotypes for hip height (HH). ONT derived GEBVs were imputed using QUILT and GLIMPSE and calculated across five coverages and five SNP panels. The different SNP reference panels were created using minor allele frequency (MAF) filters to reduce the size of the panels down from whole genome sequence SNP. The largest panel had 48,203,338 SNP and was referred to as the No MAF filter panel, while the smallest panel was referred to as the bovine high definition (HD) SNP panel and featured only the 641k SNP used to calculate the GEBVs. SNP array genotypes were from the Illumina bovine HD SNP array. The correlation for each trait between GEBVs calculated from the 35k GGP SNP array imputed to 700k and GEBVs calculated from the Illumina bovine HD SNP array are indicated by the dashed line. The colour of each bar indicates how well the ONT derived GEBV accuracies compare to the 35K SNP array accuracies. Error bars indicate 95% confidence interval of the Pearson correlation. B) Genomic prediction bias for body weight (BW), defined as** $\boldsymbol{\beta}_{\boldsymbol{2}}\boldsymbol{-1}$**, where** $\boldsymbol{\beta}_{\boldsymbol{2}}$ **is the regression coefficient of the bovine HD SNP array genomic estimated breeding value (GEBV) ~ Oxford Nanopore Technologies GEBV derived using QUILT and GLIMPSE. The prediction bias of the HD SNP array GEBVs ~ 35k SNP array GEBVs are displayed for each trait by the dotted lines, where the colour of the line corresponds to the colour of the trait in the figure legend.**


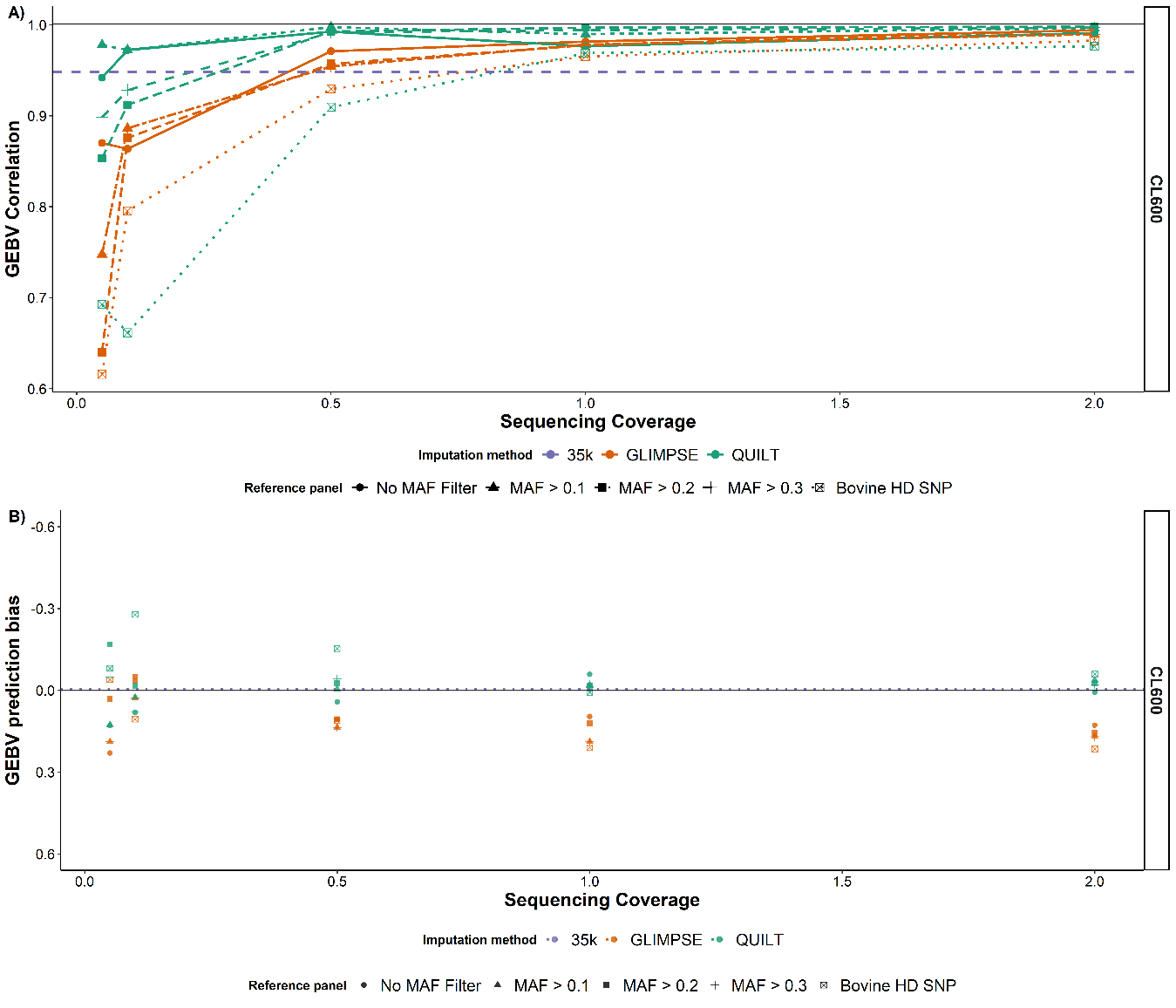


**Supplementary Figure 6. A)** **Correlations between genomic estimated breeding values (GEBVs) derived from Oxford Nanopore Technologies (ONT) sequence data and GEBVs derived from bovine HD SNP array genotypes for *corpus luteum* score (CL score). ONT derived GEBVs were imputed using QUILT and GLIMPSE and calculated across five coverages and five SNP panels. The different SNP reference panels were created using minor allele frequency (MAF) filters to reduce the size of the panels down from whole genome sequence SNP. The largest panel had 48,203,338 SNP and was referred to as the No MAF filter panel, while the smallest panel was referred to as the bovine high definition (HD) SNP panel and featured only the 641k SNP used to calculate the GEBVs. SNP array genotypes were from the Illumina bovine HD SNP array. The correlation for each trait between GEBVs calculated from the 35k GGP SNP array imputed to 700k and GEBVs calculated from the Illumina bovine HD SNP array are indicated by the dashed line. The colour of each bar indicates how well the ONT derived GEBV accuracies compare to the 35K SNP array accuracies. Error bars indicate 95% confidence interval of the Pearson correlation. B) Genomic prediction bias for body weight (BW), defined as** $\boldsymbol{\beta}_{\boldsymbol{2}}\boldsymbol{-1}$**, where** $\boldsymbol{\beta}_{\boldsymbol{2}}$ **is the regression coefficient of the bovine HD SNP array genomic estimated breeding value (GEBV) ~ Oxford Nanopore Technologies GEBV derived using QUILT and GLIMPSE. The prediction bias of the HD SNP array GEBVs ~ 35k SNP array GEBVs are displayed for each trait by the dotted lines, where the colour of the line corresponds to the colour of the trait in the figure legend.**


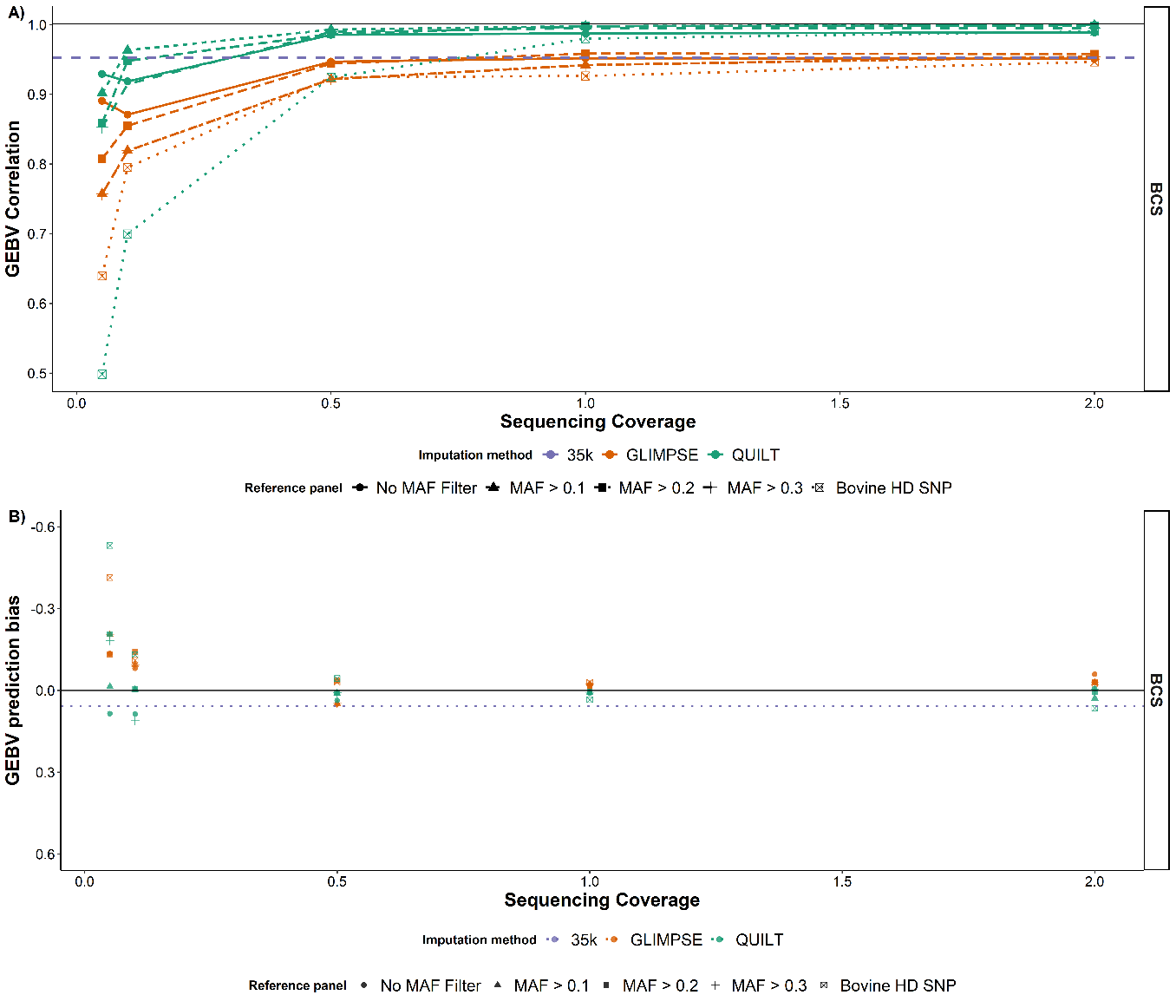


**Supplementary Figure 7. A)** **Correlations between genomic estimated breeding values (GEBVs) derived from Oxford Nanopore Technologies (ONT) sequence data and GEBVs derived from bovine HD SNP array genotypes for body condition score (BCS). ONT derived GEBVs were imputed using QUILT and GLIMPSE and calculated across five coverages and five SNP panels. The different SNP reference panels were created using minor allele frequency (MAF) filters to reduce the size of the panels down from whole genome sequence SNP. The largest panel had 48,203,338 SNP and was referred to as the No MAF filter panel, while the smallest panel was referred to as the bovine high definition (HD) SNP panel and featured only the 641k SNP used to calculate the GEBVs. SNP array genotypes were from the Illumina bovine HD SNP array. The correlation for each trait between GEBVs calculated from the 35k GGP SNP array imputed to 700k and GEBVs calculated from the Illumina bovine HD SNP array are indicated by the dashed line. The colour of each bar indicates how well the ONT derived GEBV accuracies compare to the 35K SNP array accuracies. Error bars indicate 95% confidence interval of the Pearson correlation. B) Genomic prediction bias for body weight (BW), defined as** $\boldsymbol{\beta}_{\boldsymbol{2}}\boldsymbol{-1}$**, where** $\boldsymbol{\beta}_{\boldsymbol{2}}$ **is the regression coefficient of the bovine HD SNP array genomic estimated breeding value (GEBV) ~ Oxford Nanopore Technologies GEBV derived using QUILT and GLIMPSE. The prediction bias of the HD SNP array GEBVs ~ 35k SNP array GEBVs are displayed for each trait by the dotted lines, where the colour of the line corresponds to the colour of the trait in the figure legend.**
